# Synthesis, calf thymus DNA binding, in-vitro cytotoxicity, molecular docking, and antimicrobial studies of novel metal complexes containing a 2,3-diaminopyridine derivative Schiff base

**DOI:** 10.1038/s41598-026-49189-5

**Published:** 2026-05-27

**Authors:** M. A. Helal, Rasha M. S. Shoaib, A. Z. El-Sonbati, M. A. Diab, M. A. El-Mogazy, M. M. El-Zahed, Sh. M. Morgan

**Affiliations:** 1 Chemistry Department, Faculty of Science, Damietta University, Damietta, Egypt; 2 Food and Dairy Sciences and Technology Department, Faculty of Environmental Agricultural Sciences, Arish University, Arish, North Sinai Egypt; 3 Botany and Microbiology Department, Faculty of Science, Damietta University, Damietta, Egypt; 4 Environmental Monitoring Laboratory, Ministry of Health, Port Said, Egypt; 5 Health Technical Institute, Ministry of Health, Port Said, Egypt

**Keywords:** Metal complexes, Calf thymus DNA binding, Molecular docking, Biological activity, X-Ray diffraction analysis, Biochemistry, Cancer, Chemical biology, Chemistry, Computational biology and bioinformatics, Drug discovery

## Abstract

In this work, a tetradentate ONNO Schiff base ligand, namely 4,4'-[(1*E*,1*`E*)-(pyridine-2,3-diyl)bis(azanylylidene)bis(methanylylidene)bis (benzene-1,3-diol] (H_2_L) was prepared and characterized. Cu(II), Co(II), Mn(II), and Ni(II) complexes have an octahedral geometry. Pd(II) complex exhibits diamagnetic behavior and attains stability in square planar locations. The complexes are non-electrolytes, according to the molar conductivity investigations. By means of two phenolic OH and two azomethine nitrogen, the ligand is thought to be tetradentate and coordinated with Co(II) and Ni(II) ions, according to spectral data. Cu(II), Mn(II), and Pd(II) complexes, on the other hand, were coordinated by two phenolic oxygen and two azomethine nitrogen with deprotonation of OH groups. Antimicrobial activities, ligands, and metal complexes were screened against bacteria, such as *E. faecalis*, *Salmonella* sp., *B. cereus,* and *S. epidermidis*, and fungi, such as *A. flavus*, *A. solani*, and *C. albicans*. Notably, all synthesized compounds demonstrated good results of inhibition against the chosen pathogenic fungi compared to their antibacterial properties. Molecular docking was utilized to explore possible interactions between the studied compounds (H_2_L and complexes) and the breast cancer cell line. The abovementioned compounds were proven to have potential anticancer effects against protein receptors (PDB IDs: 1QNT, 3FC2, and 4AJY). The least binding energy was used to predict the receptor’s binding site with compounds, offering a better understanding of the process. Furthermore, the MTT test was employed to assess the in vitro cytotoxicity of the compounds against the MCF-7 cancer cell line in order to assess the anticancer characteristics of the Schiff base and its complexes. The findings demonstrated that each combination has exceptional antitumor activity.

**Supplementary Information:**

The online version contains supplementary material available at 10.1038/s41598-026-49189-5.

## Introduction

Schiff base compounds and their complexes are important, applicable compounds owing to their remarkable donor qualities, ease of synthesis, and high solubility in ubiquitous solvents^1–3^. These Schiff bases containing azomethine groups have important antifungal, antibacterial, food, leather, textile, medical, and cosmetic characteristics. Because Schiff base compounds may form complexes with transition metal ions that can combine with DNA molecules through coordinating interactions with their nitrogen atoms to create a chelate ring, they have shown promise as antibacterial agents in the medical area. Because of their coherent pattern and manufacture in the field of coordination chemistry, as well as the distinguished purposes in bioinorganic chemistry and as functional materials, the chemistry of transition metals with Schiff base compounds has gained a lot of interest. As antifungal, antibacterial, anticancer, and antibiotic drugs, transition metal-containing nitrogen donors are used in a variety of applications^3–6^. Several authors have addressed this type of tetradentate Schiff base since the structure of Schiff bases comprises two nitrogen and two oxygen atoms that provide an interesting manner to coordinate with metal ions in four active sites^5–7^.

Particular attention is claimed by Schiff base transition metal complexes made of benzohydrazides and naphthaldehyde because of their ability to chelate and their structural flexibility. They are widely used in biological activities, such as cytotoxicity, antioxidants, anti-inflammation, and antimicrobials^8–11^. Bioinorganic chemists are now investigating the potential therapeutic applications of transition metals like Cu(II), Co(II), Mn(II), and Ni(II) due to their importance in medicine^12,13^. Since DNA is the ultimate biological target of all chemotherapeutics, understanding how metallo-drugs interact with it is a crucial first step in the quest for new cytotoxic treatments at the cellular level^14^.

Cancer is a large class of disorders characterized by abnormal cell proliferation that can cause it to invade or spread to other body areas. The MCF-7, a human breast cancer cell line, is widely used for research on anticancer activities and medication development, and it is an efficient model for breast cancer. From this perspective, the present study is undertaken to explore and throw more light on the antimicrobial properties and in vitro anticancer activities of synthesized metal complexes against the MCF-7cell line.

The preparation and characterization of a tetradentate ligand are highlighted in this study. The ligand was obtained by condensation of 2,3-diaminopyridine and 2,4-dihydroxybenzaldehyde, respectively, in ethanol at a 1:2 molar ratio, resulting in Schiff base 4,4'-[(1*E*,1*`E*)-(pyridine-2,3-diyl)bis(azanylylidene)bis(methanylylidene)bis(benzene-1,3-diol] (H_2_L). Novel complexes of Cu(II), Co(II), Ni(II), Mn(II), and Pd(II) with Schiff base ligand (H_2_L) were prepared. Coupling structural and spectroscopic methods allowed for the clarification of the structures of the produced compounds. The antibacterial and antifungal activity of the produced complexes was also evaluated against a variety of microbial strains. In addition, molecular docking was utilized to explore possible interactions between the studied compounds (H2L and complexes) and the breast cancer cell line. The formed compounds were proven to have potential anticancer effects against protein receptors (PDB IDs: 1QNT, 3FC2, and 4AJY). The least binding energy was used to predict the receptor’s binding site with compounds, offering a better understanding of the process.

## Experimental

All chemicals used in this study were of chemically high pure great from (BDH). Water was double-distilled. Every technique, tool, and measurement is used in accordance with the previously published reports^15–25^.

### Synthesis of the ligand (H_2_L)

**Scheme 1:**
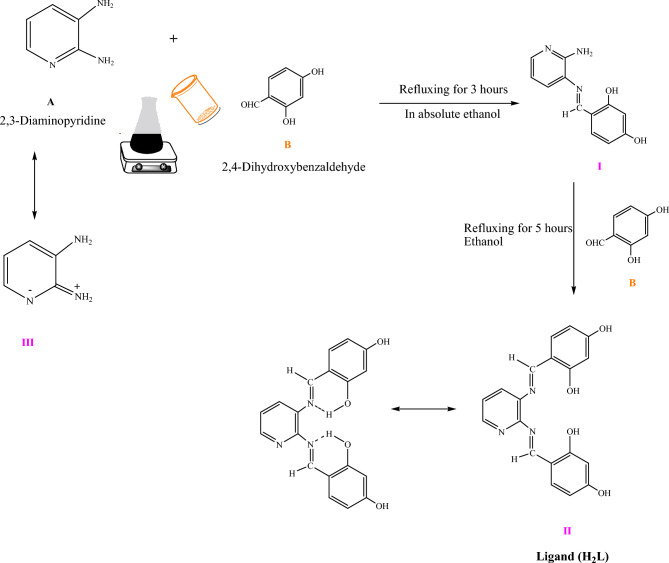
Synthesis of Schiff base ligand (H_2_L).

Because 2,3-diaminopyridine has two different condensation sites, one might expect two mono Schiff bases to be formed by 1:1 condensation; however, only compound** I** was obtained (Scheme 1). The lower reactivity of the 2-amino group with respect to the 3-amino group may be attributed to the contribution of the resonance structure **III**^26^ (Scheme 1). The product was purified by recrystallization from hot ethanol, and then dried in a vacuum desiccator over anhydrous P_2_O_5_. Yield 80%; solid. Color yellowish orange, M.P. 172 °C: Anal. Calcd. for C_19_H_15_N_3_O_4_ (%): C, 65.33; H, 4.30; N, 12.03; Found (%): C, 65.21; H, 4.19; N, 11.88. FT-IR (υ, cm^−1^): azomethine (C = N) 1606, N-pyridine ring 1108, amino group (NH_2_) bending 616. UV–Vis (λ_max_, cm^−1^): 36,260 & 32,460 cm^−1^ (π–π* of benzene rings) and 29,675 (π–π* of azomethine C = N).

### Preparation of complexes

**Scheme 2:**
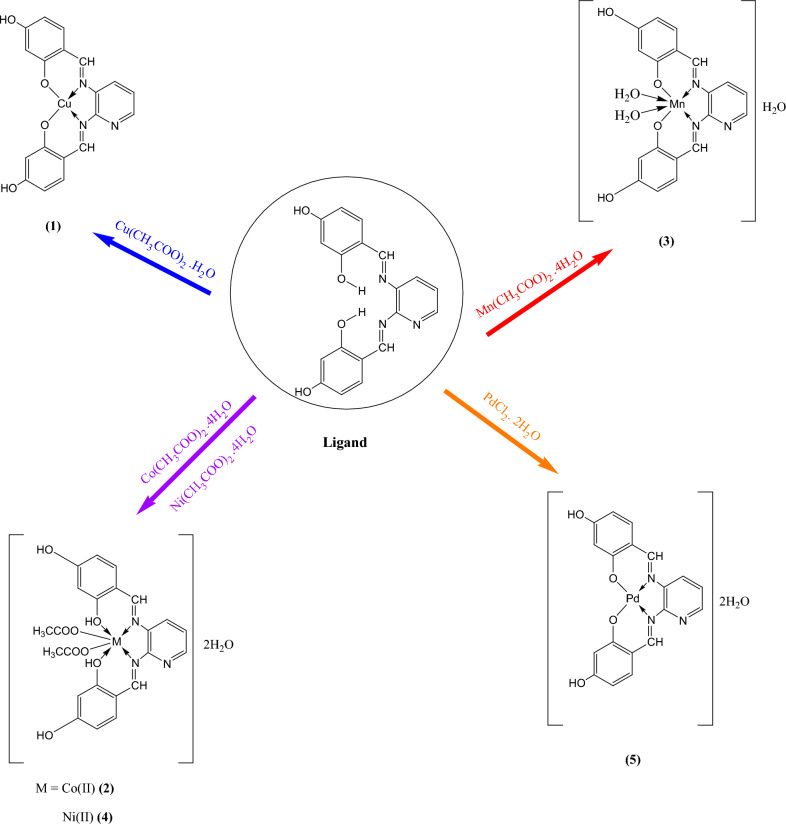
The structures of Schiff base metal complexes.

All operations leading to the formation of the complexes and isolation of the final product were carried out using degassed solutions and solvents and under nitrogen that had been freed from oxygen. The general method to obtain the complexes consists of adding, under continuous stirring, to an ethanolic solution of metal (II) acetate salts or palladium chloride, a ligand solution **II** in a molar ratio (1M:1L). The formation of the complex compound takes place immediately. Stirring was continued for 2 h at room temperature. The mixture was refluxed for 2 h, and the final precipitate (Scheme 2) was filtered, washed with a little EtOH, and dried in vacuo. The purity of the complexes was checked by taking thin layer chromatography (TLC).

4,4'-[(1*E*,1*`E*)-(Pyridine-2,3-diyl)bis(azanylylidene)bis(methanylylidene)bis (benzene-1,3-diol] (H_2_L) ligand (Scheme 1) and its complexes were made in accordance with a previously reported ^7,27,28^. Table 1 lists the analytical data of complexes, while Scheme 2 displays the complex structures. The formation of these complexes may be proposed according to the following equation:

**Table 1:** Elemental analysis data of complexes** (1–5)**.

Compd.^a^	Formulae	% Found(Calcd.)			
		C	H	N	M
**(1)**	[Cu(L)]	55.35 (55.54)	3.09 (3.17)	10.03 (10.21)	15.35 (15.48)
**(2)**	[Co(H_2_L)(CH_3_COO)_2_] 2H_2_O	49.08 (49.12)	3.56 (3.74)	7.25 (7.47)	10.29 10.49)
**(3)**	[Mn(L)(OH_2_)_2_] H_2_O	49.89 (50.01)	3.63 (3.73)	9.04 (9.21)	11.98 (12.05)
**(4)**	[Ni(H_2_L)( CH_3_COO)_2_] 2H_2_O	49.05 (49.14)	3.62 (3.74)	7.29 (7.48)	1022 (10.45)
**(5)**	[Pd(L)] 2H_2_O	46.37 (46.59)	2.48 (2.66)	8.27 (8.58)	21.55 (21.74)

^a^Numbers as given in Scheme 2.

H_2_L + Cu(CH_3_COO)_2_.H_2_O → [Cu(L)] + 2CH_3_COOH + H_2_O.

H_2_L + Co(CH_3_COO)_2_.4H_2_O → [Co(H_2_L)(CH_3_COO)_2_] 2H_2_O + 2H_2_O.

H_2_L + Ni(CH_3_COO)_2_.4H_2_O → [Ni(H_2_L)( CH_3_COO)_2_] 2H_2_O + 2H_2_O.

H_2_L + Mn(CH_3_COO)_2_.4H_2_O → [Mn(L)(OH_2_)_2_] H_2_O + 2CH_3_COOH + H_2_O.

H_2_L + PdCl_2_.2H_2_O → [Pd(L)] 2H_2_O + 2HCl.

### Antimicrobial potential

*Staphylococcus epidermidis*, *Bacillus cereus*, *Salmonella* sp., *Enterococcus faecalis*, *Aspergillus flavus*, *Alternaria solani*, and *Candida albicans* were kindly provided by the Microbiology Lab, Botany and Microbiology Department, Faculty of Science, Damietta University. For the subculturing of bacterial and fungal strains, nutrient and DOX agar media were utilized, respectively. The antimicrobial activity was tested using the agar well diffusion method, minimum inhibition concentration (MIC), minimum microbicidal concentration (MMC), time-kill assay, and enzymatic antioxidant assay tests. The experiments were performed in triplicate.

#### Agar well diffusion method

A 0.5 McFarland standard spore suspension from all examined microbial strains was made, inoculated, and thoroughly mixed with cooled molten Muller-Hinton agar (MHA) media before being transferred into Petri dishes. 100 µl of different concentrations (50, 100, and 150 µg/ml) from the tested compounds were added to each of the 1 centiliter wells that were made in each Petri plate^29^. Standard antibiotics were utilized, namely terconazole and ampicillin, as positive controls. For 48 h or five days, plates were incubated at 37 °C or 30 °C for bacterial and fungal strains, respectively. Lastly, measurements were made in millimeters of the inhibitory zones around every well^6^.

#### Microbial cell viability test

The MIC of the ligand (H_2_L) and the metal complexes was investigated using the broth dilution technique assay to assess microbial cell viability^30^. Each microbial strain’s 0.5 McFarland standard was added to 96-well plates containing Muller-Hinton broth (MHB) growth media supplemented with varying concentrations of the ligand or the metal complexes. As a control, untreated microbial cells were cultured in medium. A microplate plate reader was used to assess absorbance at 600 nm for bacteria or fungi, respectively, following 48 h or 5 days of incubation.

#### Minimum microbicidal concentration

Additionally, the compound’s MMC was investigated. The mixture was transferred into sterile Petri dishes after thoroughly mixing the medium supplemented with the MIC from the compound and inoculating a 0.5 McFarland standard from each microbial strain. For 48 h or 5 days, the inoculated agar plates were cultured for bacteria or fungi. Without any visible colonial growth plates, the MMC values of the antibacterial or antifungal activity were calculated^31^.

#### Time-kill assay

Fresh MHB containing MMC of the ligand or the metal complexes was inoculated with 0.5 McFarland standard of each microbial strain and incubated for 24 h at 37 °C. From 0 to 24 h, the number of surviving microbial cells was counted every 3 h. The surviving colonies were enumerated and expressed as CFU/ml after 100 μl of the samples were sub-cultured on MHA plates. A reduction of ≥ 3 log_10_ CFU/ml from the original in oculum was considered a microbicidal effect^32^.

#### Enzymatic antioxidant assay

The activity of peroxidase (POX) and catalase (CAT) for microbial strains treated with ligand and its complexes was evaluated^33^. POX activity was measured by combining the MMC from each chemical with the enzymatic reaction (4-aminoantipyrine, potassium phosphate buffer, 2,4-DCP, and H_2_O_2_) for one minute. In order to assess CAT activity, drug MICs were mixed with phosphate buffer and H_2_O_2_. Using spectrophotometry, the oxidation-induced rise in absorbance was measured at 510 and 415 nm in relation to the untreated microorganisms as a control. Moles of H_2_O_2_ per liter were equivalent to one unit ml^-1^ of enzyme.

### DNA-binding instruments

The binding properties of the ligand and its metal complexes to calf thymus DNA (CT-DNA) have been studied using electronic absorption spectroscopy (EAS)^6^. The stock solution of CT-DNA was prepared in 5 mMTris–HCl/50 mM NaCl buffer (pH = 7.2), which a ratio of UV absorbance at 260 and 280 nm (A_260_/A_280_) of ca. 1.8–1.9, indicating that the DNA was sufficiently free of protein and the concentration was determined by UV absorbance at 260 nm (Ɛ = 6600 M^−1^ cm^−1^). Electronic absorption spectra were carried out using 1 cm quartz cuvettes at 25 °C by fixing the concentration of ligand or complexes (1.00 × 10^−3^ mol L^−1^), while gradually increasing the concentration of CT-DNA (0.00 to 1.30 × 10^−4^ mol L^−1^). An equal amount of CT-DNA was added to both the compound solutions and the reference buffer solution to eliminate the absorbance of CT-DNA itself.

Electronic absorption spectroscopy was used to examine the binding characteristics of the ligand and its metal complexes to CT-DNA. The intrinsic binding constant (K_b_) of the ligand and its metal complexes with CT-DNA was determined using the following equation^6,21^:

$$\frac{[DNA]}{{(\varepsilon_{a} - \varepsilon_{f} )}} = \frac{[DNA]}{{(\varepsilon_{b} - \varepsilon_{f} )}} + \frac{1}{{K_{b} (\varepsilon_{a} - \varepsilon_{f} )}}$$

where b is the compound’s molar extinction coefficient when fully bonded to DNA, є_a_ is the molar extinction coefficient of the free compound in solution, and [DNA] is the concentration of CT-DNA in base pairs. Additionally, є_b_ is the molar extinction coefficient observed for the A_obs_/[compound] at the given DNA concentration. Plotting [DNA]/(є_a_–є_f_) against [DNA] yields K_b_, which is determined by dividing the slope by the intercept.

### Cytotoxicity study

The human breast cancer (MCF-7) strain was donated by the American Type Culture Collection (ATCC, Manassas, VA, USA). 10% FBS was added to DMEM media during the cell culture process. They were kept at a regulated temperature of 37 °C in an incubator with 5% CO_2_. Following their suspensions in phosphate buffer saline (PBS), cultures containing cells were collected, counted using a hemocytometer, and the MTT assay was used to determine the cultures’ viability.

The cytotoxicity of the metal complexes was assessed using the MTT test. The technique is based on the fact that live cells can reduce 3-(4,5-dimethyl-2-thiazol)-2, 5-diphenyl-2H-tetrazolium bromide dye. In triplicate, 8 × 10^3^ MCF-7 cells were seeded onto 96-well plates. The cells were then attached to the plate by incubating them for 24 h at 37 °C with 5% CO_2_. The next day, successive amounts of ligand and its complexes (0.01, 0.1, 1, 10, 100 μM) were produced at a constant concentration in order to treat the cells. As a positive control, doxorubicin was used, and the carrier solvent (0.1% DMSO) was applied to control cells. Both treated and untreated cells were grown for 48 h at 37 °C in an atmosphere with 5% CO_2_ after the complexes were added. After the incubation period, the Vybrant® MTT Cell Proliferation experiment Kit, cat no. M6494 (Thermo Fisher, Germany) was used to perform the cell cytotoxicity experiment. 20 μL of MTT solution (1 mg/mL) was added to the cell culture medium, and it was then incubated for four hours at 37 °C. The MTT solution was then disposed of, and the wells were filled with 100 μL of sodium dodecyl sulfate with hydrochloric acid (SDS-HCL). After the cell proliferation assay, the relationship between the log dosages of the inhibitor and the normalized response was represented by the plotting of the XY curve, and the best-fit point was determined through the use of linear regression analysis. The optical density at 570 nm was measured using a spectrophotometer (ELx 800; Bio-Tek Instruments Inc., Winooski, VT, USA) to assess the vitality of the cells. Using GraphPad Prism 9, version 9.1.0 (221), the half-maximal stimulatory concentration (IC_50_) that results in a 50% inhibition of cell growth was determined. Based on concentration–response curves of examined cellular metabolic activity that were standardized to untreated MCF7 cells, the IC_50_ for each group was determined. Experiments were run three times.

### Molecular docking

The ligands were selected using molecular docking, a well-known and adaptable in silico method that was used to anticipate the binding interactions between each protein. Using Molecular Orbital Environment (MOE) software, we performed molecular docking studies of ligand and Cu(II), Co(II), Ni(II), Mn(II), and Pd(II) complexes to examine the binding modes between the compounds and the targeted enzymes (PDB IDs: 1QNT, 3FC2, and 4AJY). The Protein Data Bank provided the specific proteins that were utilized^6^.

1QNT: This protein is associated with kinase activity, a key driver of cell proliferation and survival in breast cancer. Kinases often regulate signaling pathways like PI3K/AKT and MAPK, which are frequently dysregulated in MCF-7 cells. Inhibiting kinase activity can disrupt uncontrolled cell division and induce apoptosis.

3FC2: Identified as a receptor tyrosine kinase (RTK), 3FC2 plays a critical role in metastasis and angiogenesis. RTKs are known to activate pathways that promote tumor growth and resistance to therapy. Targeting 3FC2 could block these pro-oncogenic signals.

4AJY: This protein is linked to DNA repair mechanisms, particularly in homologous recombination. Cancer cells often exploit such pathways to survive genotoxic stress (e.g., chemotherapy). Inhibiting 4AJY may sensitize MCF-7 cells to DNA-damaging agents or induce synthetic lethality.

For MOE to show, the structures of the investigated drug have been established using ChemDraw Ultra 12.02 and stored as MDL files (".sdf").

Originally, the integrated “Quickprep” feature was used to prepare the protein files. The recovered proteins underwent manual removal of water molecules and their corresponding ligands. Placement was done using Triangle Matcher, scoring was done with London dG, the docking site was set to fake atoms, and the resultant protein active site file was loaded. For each tested chemical, the scoring system was modified to GBVI/WSA dG, and the refining procedure was altered to a rigid receptor in order to determine the top 5 poses out of 100 possible postures. The dock calculations were carried out automatically, and the molecular library was imported and made ready for use in the MOE database.

## Results and discussion

### Chemistry

2,4-Dihydroxybenzaldehyde and 2,3-diaminopyrine were directly condensed to create the Schiff base, H_2_L (Scheme 1). The stoichiometry of 4,4'-[(1*E*,1*`E*)-(pyridine-2,3-diyl)bis(azanylylidene)bis(methanylylidene)bis (benzene-1,3-diol] (H_2_L) was in good agreement with the elemental analysis data that were obtained. A generic technique that used an ethanolic solution of metal ions and a solution of ligand in absolute ethanol in a 1:1 molar ratio was used to create the Cu(II), Co(II), Mn(II), Ni(II) acetate, and PdCl_2_^33^. Metal(II) complexes coordination resulted in the formation of five and six-chelate rings to the tetradentate Schiff base (ONNO). Higher stability of the compounds is determined by their ring forms^34^. Only the powder form of the ligand and its complexes were generated, making them unsuitable for single X-ray diffraction examination. Subsequent efforts to obtain crystals failed. No specific structures are reported because it was not possible to separate single crystals of the compounds from any organic solution. However, as can be shown below, the analytical and spectroscopic data allow us to predict potential structures. Complexes matching to the general formulas [Cu(L)](brown) **(1)**, [Co(H_2_L)(CH_3_COO)_2_]2H_2_O (dark brownish) **(2)**, [Mn(L)(H_2_O)_2_]H_2_O **(**yellowish brown**) (3)**, [Ni(H_2_L)(CH_3_COO)_2_]2H_2_O (dark brown) **(4)** and [Pd(L)]2H_2_O (pale brown) **(5)** were produced when the ligand (H_2_L) interacted with metal(II) salts. Every complex has a high melting point and is colored. Only DMF and DMSO will dissolve them; they are insoluble in other organic solvents. In DMSO (10^–3^ M solution at 25 °C), the soluble complexes molar conductance values. It was discovered that all of the complexes** (1–5)** were non-electrolytes, as shown by the range of 13–18 Ω^-1^ cm^2^ mol^-1^^35^.

The purity and stability of the compounds are established by the observance of high melting/decomposition temperature. The complexes showed a higher melting point (˃ 300 °C) than the ligand (172 °C), which may be due to the inter-molecular bonding as a result of the metallic lattice, and an increase in molecular weight indicates that the synthesized compounds are presumed pure^36^. An appreciable percentage yield of all new compounds was obtained, which ranged from 65 to 78%.

### FT-IR spectra

The FTIR spectra provide valuable information regarding the nature of the functional group attached to the metal atom.

**Fig. 1:**
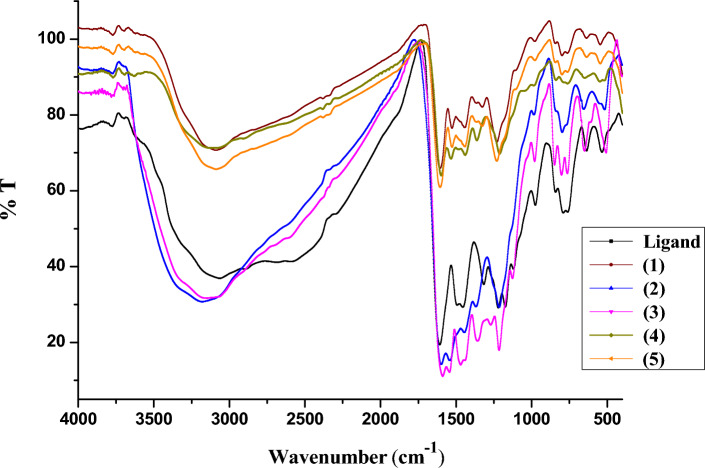
FTIR spectra of the ligand and its metal complexes.

The solid-state properties of the Schiff base (H_2_L, Scheme 1) were examined by FT-IR spectroscopy. The spectrum of H_2_L exhibits a strong band at 1606 cm^-1^, which is characteristic of the υ(HC = N) group. It is expected that coordination of the nitrogen centre to the metal ion would reduce the electron density in the azomethine link and thus shift the υ(HC = N) to a lower wave number^37,38^. In the FT-IR spectra of the complexes, this bend is shifted to the region at 1587–1610 cm^-1^^39–42^. An intense band at 1230 cm^-1^ in H_2_L has been assigned to the phenolic υ(C–O) stretch. In complexes, this band is shifted to higher frequencies, indicating the coordination of H_2_L through the deprotonated phenolic (C_2_–O)^43^. These data have been further supported by the disappearance of the broad band at 3341 cm^-1^ attributed to υ(C_2_–OH) for complexes **(1, 3** & **5)**. Whereas the final band was moved to a lower frequency in the metal complexes **(2** &** 4)**, suggesting that coordination occurs in the phenolic hydroxyl groups (C_2_–OH) of H_2_L. Two bands in the range 657–545 and 515–505 cm^-1^ were assigned to υ(M–O) and υ(M- N), respectively^23,38,44^. The FT IR spectra of the ligand and its metal complexes are shown in Fig. 1. The presence of coordinated water in complex **(3)** is suggested by the new broad band seen in complexes at ≈3351–3170 cm^-1^^45–51^. Acetate coordination in a monodentate form is confirmed by the υ_asy_(COO^-^) and υ_sym_(COO^-^) bands in complexes **(2** &** 4)** that vary by more than 200 cm^-1^^42,45^. According to the previously reported findings, the ligand interacts with the ONNO donor positions via tetradentate to the metal.

According to the previously described results and the examination of complexes (**1**, **2**, and **4**), their chelation seems to have caused the C = N band to be moved to lower wavenumbers. However, it is important to remember that the amount that the frequency shifts alter depends on the ligand and metal ion composition. This was mainly explained by the change in the position of the vibrating dipole and the strength of the electrostatic field of the metal ion. For metal ions with the same charge, the distance from the coordinated groups is the main factor affecting band shifts. Issa et al.^52^ used the amplitude of frequency shifts to calculate the distance between the coordinated groups and the metal ion, which is about equal to the length of the coordination bond. The IR spectra of organic ligands change when they are coupled to metal ions in a manner akin to that of their adsorption on a salt substrate^28,53^. The coordination bond length (r) may be computed using the relation below:

$$\Delta {{\boldsymbol{\upupsilon}}} \, = \left( {{32 }\pi /{\mathrm{a}}^{{2}} } \right)[\alpha \, \left( {\upsilon_{{{\mathrm{x}} = {\text{y }}}} - \upsilon_{{{\mathrm{x}} - {\mathrm{y}}}} } \right){\mathrm{exp}}\left( { - {2}\pi \surd {\mathrm{2r}}/\alpha } \right]$$

where α = bond polarisability and Δυ = shift in the oscillator frequency (υ_compound_ − υ_complex_). The α values were computed from published data for complexes with structures similar to those of the present complexes. a is the metal salt lattice constant, and υ_x-y_ is the oscillator’s frequency with a double bond. I is the oscillator’s length coordinated to the metal ion, and υ_x-y_ is its frequency when operating with a single bond.

The r values for the bond between the metal and the nitrogen atom of the (HC = N) group were calculated using these data. The ligand-transition metal complexes Ni(II) (3.23), Co(II) (3.05), and Cu(II) (3.50) have the following sequence of estimated coordination bond lengths (r). The M–N frequencies and coordination bond lengths (r) for the metal complexes that are being studied. The coordination bond lengths and M–N frequencies of the ligand transition metal complexes are as follows: Cu(II) ˃ Ni(II) ˃ Co(II). The Co(II) ion’s larger ionic radius than that of Cu(II) or Ni(II) may be the cause of the cobalt complex’s shorter coordination bond length.

### ^1^H NMR and ^13^C NMR spectra

**Fig. 2:**
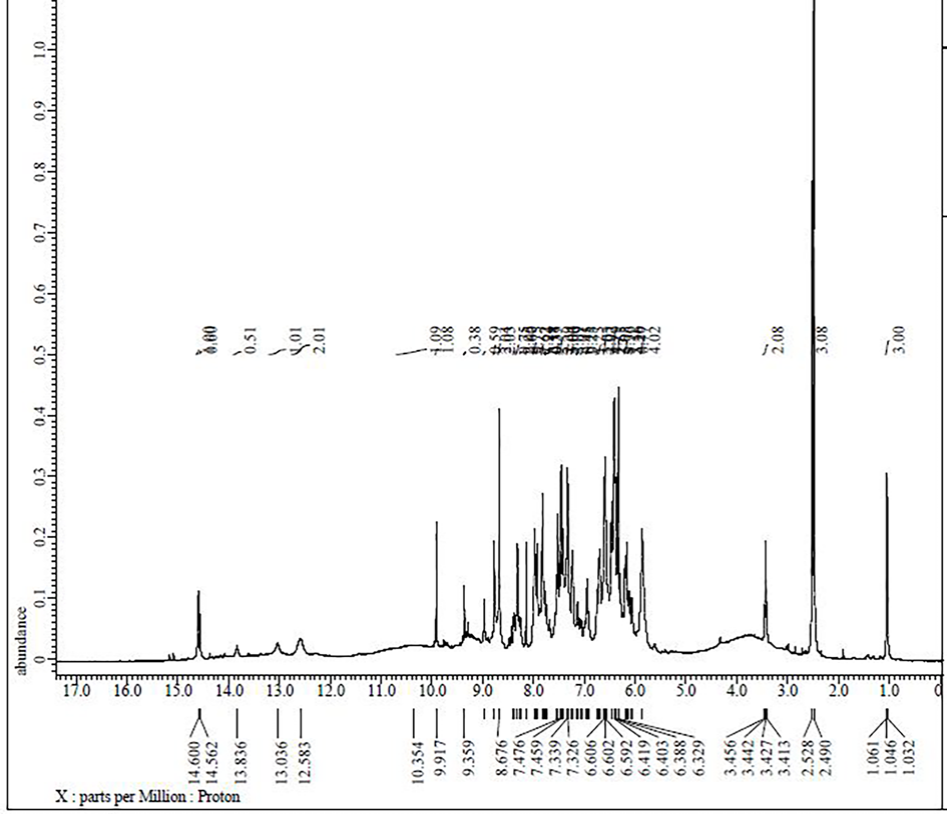
The ^1^H NMR spectrum of Schiff base ligand (H_2_L).

**Fig. 3:**
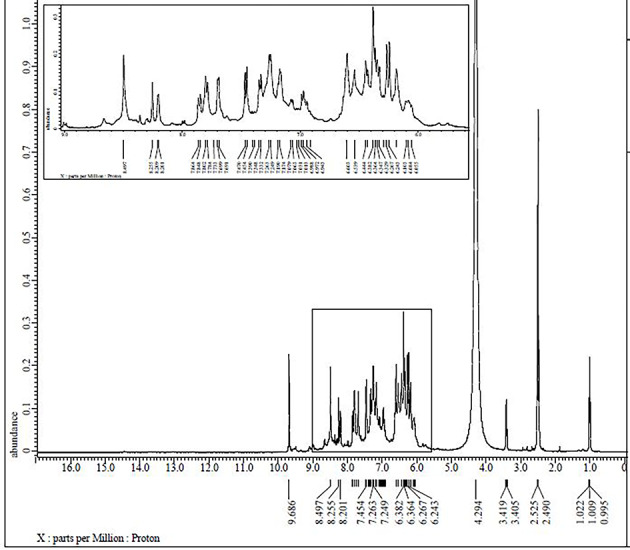
The ^1^H NMR spectrum of Schiff base ligand (H_2_L) with D_2_O.

**Fig. 4:**
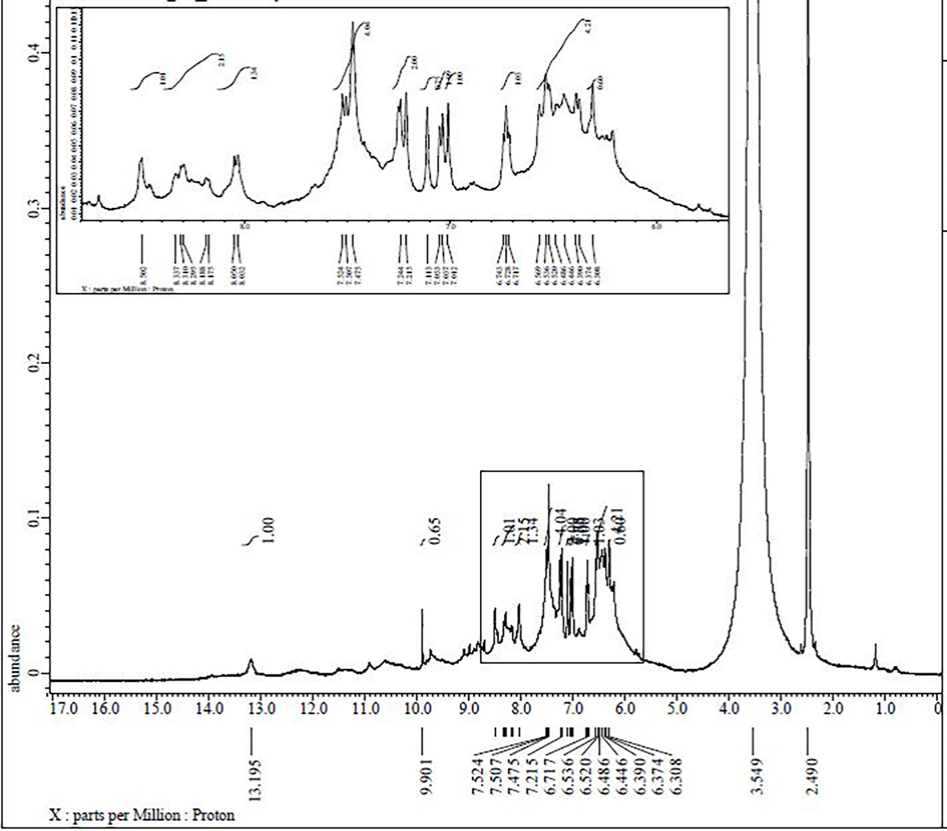
The ^1^H NMR spectrum of Pd(II) complex **(5)**.

**Fig. 5:**
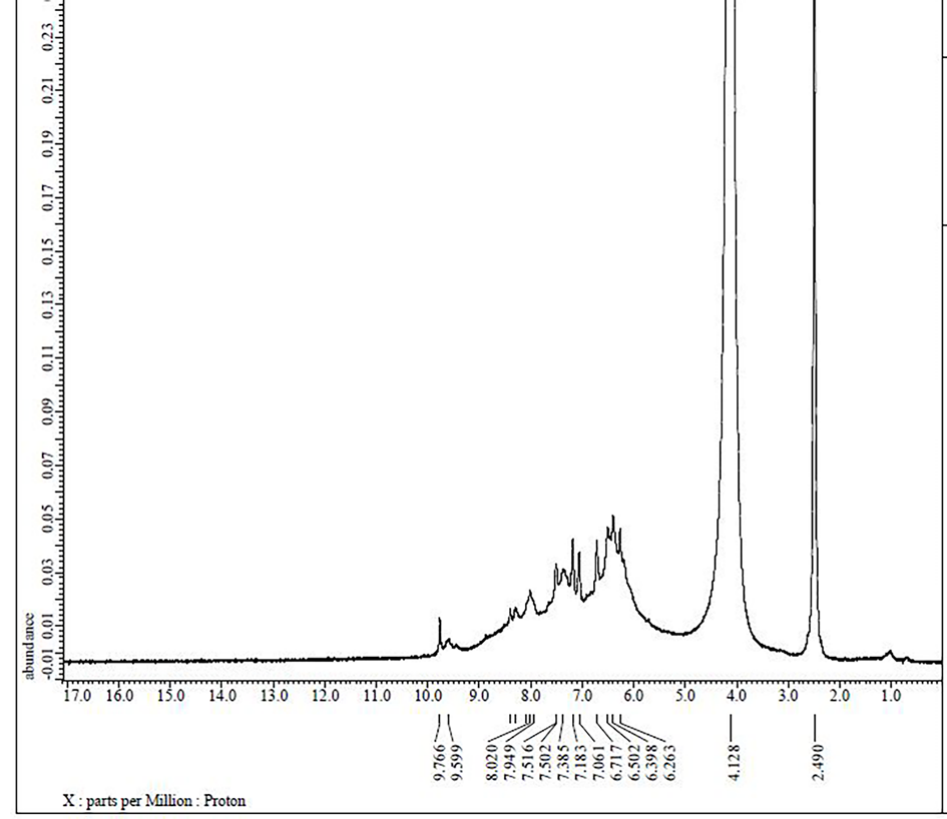
The ^1^H NMR spectrum of Pd(II) complex **(5)** with D_2_O.

The ^1^H NMR and ^13^C NMR spectra of the ligand and Pd(II) complex **(5)** were recorded in DMSO-d_6_ as shown in Figs. 2, 3, 4 and 5. The ^1^H and ^13^C NMR chemical shifts for the ligand and Pd(II) complex **(5)** are listed in Tables 2 and 3. Characteristic ^1^H-NMR peaks of ligand (Fig. 2) exhibits at ~ 9.917–9.359 ppm δ[N = CH, for two protons, [H7 & H12] groups supporting positive evidence of the formed Schiff base (H_2_L)^37^. At ~ 14.600–14.562 ppm δ[OH, for two protons, H1 & H2], two deshielded signals of two protons appeared and were assigned to the phenolic –OH presented at *ortho* and *para* positions of azomethine groups. The intramolecular and intermolecular hydrogen connections between OH groups are responsible for the singlet 2-OH groups that are located on the higher field side. The other 13.086—13.836 ppm δ [4-OH] for two protons, [H4 & H9] groups. Addition of D_2_O to the previous solution results in diminishing the signals due to proton exchange (Fig. 3). Three phenylenes with the numbers H13, H14, and H15, respectively, were responsible for a multiplet with three protons centered at δ 7.459, 7.476 and 8.670 ppm. The phenolic ring proton present ortho to azomethine was given a doublet of doublets with a center at δ ~ 7.336 [labeled as [H6 & H11]] in structure in Table 2. A multiplet for two protons centered at δ 6.592, 4.419 ppm was linked to the azomethine [H5 & H10] phenolic ring proton. Two protons were detected as a singlet at δ (6.606 and 6.602) ppm, which was attributed to a proton from a phenolic ring sandwiched between two hydroxyl groups [H3 & H8]. The singlet observed at δ (14.600 and 14.562) ppm in H_2_L is absent in complex (**5**) (Fig. 4), indicating deprotonation of phenolic groups before coordination to the metal ion. The two other 4-OH protons were shifted to a lower field. The imine proton shifted to a lower field in complex **(5)**, indicating the coordination of the ligand with the central metal ion, due to the strong shielding effect of the hydroxyl groups^54,55^. The values of aryl protons were also shifted to a lower field, indicating chelation of the ligand.

**Table 2:** ^1^H NMR chemical shifts of ligand (H_2_L) and metal complex **(5)** in DMSO-d_6_, δ ppm.

	
Atom position	δ, ppm	Atom position	δ, ppm
H1^a^	14.600	H1^b^	–
H2^a^	14.562	H2^b^	–
H7	9.917	H7	9.901
H12	9.359	H12	9.901
H6	7.339	H6	7.524
H11	7.336	H11	7.507
H13	7.459	H13	6.536
H14	7.476	H14	7.215
H15	8.676	H15	6.717
H3	6.606	H3	6.536
H8	6.602	H8	6.486
H5	6.592	H5	6.390
H10	4.419	H10	6.374
H4	13.086	H4^c^	13.195
H9	13.836	H9^c^	13.195

^a^The signals at 14.600, 14.562 ppm are attributed to the phenolic protons (H1 and H2), where their engagement in the intramolecular hydrogen bond interaction (O–H––-N) shifts their signals upfield.

^b^The absence of the phenolic δ(OH) proton signals in the complexes indicates the coordination by phenolic oxygen to the metal ion after deprotonation.

^c^Please see Fig. 5. The broad singlet around 10.354 ppm may be due to the proton of EtOH as a solvent.

**Table 3:** ^13^C NMR chemical shifts of ligand (H_2_L) and metal complex **(5)** in DMSO-d_6_, δ ppm.

	
Atom position	δ, ppm	Atom position	δ, ppm
C1	190.978	C1	191.007
C8	190.978	C8	191.007
C3	165.682	C3	184.139
C10	165.244	C10	184.139
C7	163.822	C7	165.225
C14	163.317	C14	165.225
C16	163.260	C16	163.307
C15	163.088	C15	144.650
C19	162.478	C19	133.481
C18	161.533	C18	132.861
C17	162.182	C17	124.916
C5	160.723	C5	122.150
C12	160.408	C12	119.841
C6	159.139	C6	113.687
C13	157.747	C13	113.687
C4	156.602	C4	109.187
C11	156.287	C11	108.710
C2	154.017	C2	102.873
C9	151.051	C9	102.234

**Fig. 6:**
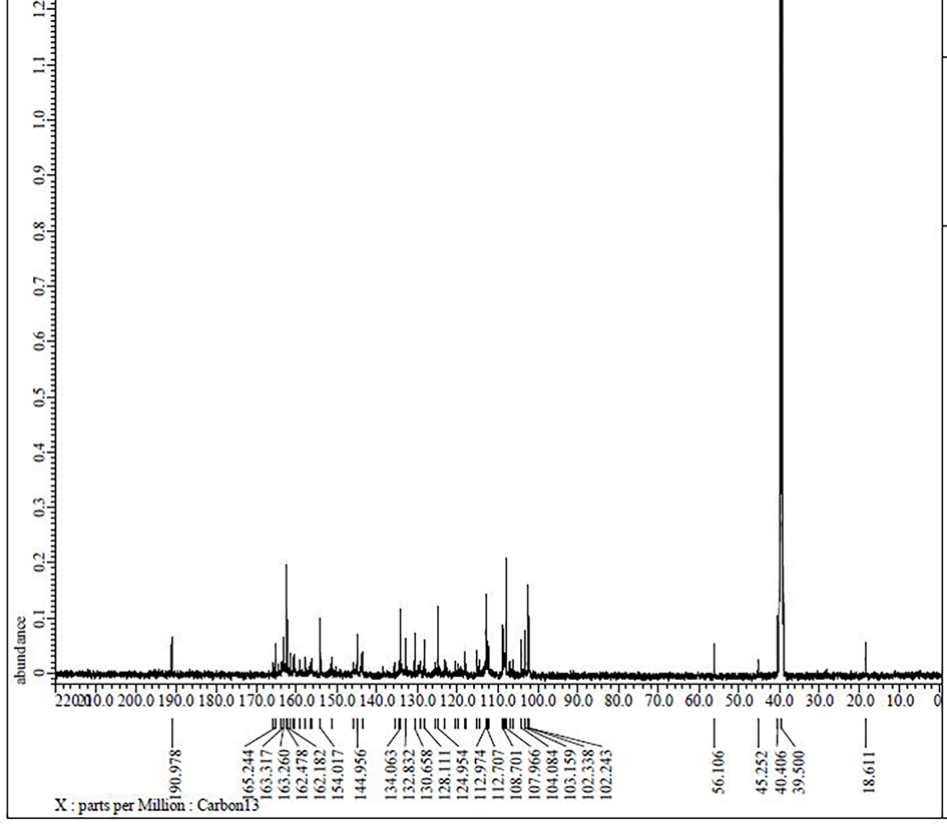
The ^13^C NMR spectrum of Schiff base ligand (H_2_L).

**Fig. 7:**
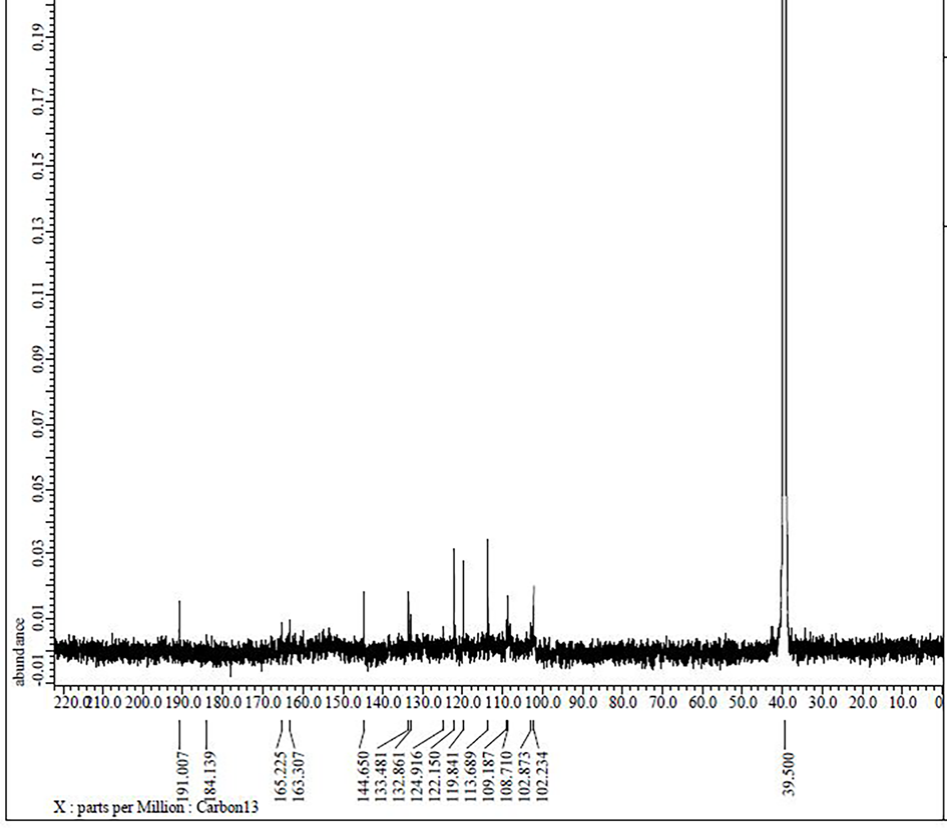
The ^13^C NMR spectrum of Pd(II) complex **(5)**.

More detailed information about the structure of the ligand was provided by ^13^C NMR spectral data (Fig. 6 and Table 3), which reveals the strong signal at δ ~ 163.832 and 163.317 ppm, which was assigned to the presence of imine (C7 and C14, > C = N–) carbons. A signal at δ ~ 190.978 ppm was assigned to carbon (C1 and C8) connected to a hydroxyl group. The signals were displayed by the aromatic carbons of the phenolic moiety range and the phenylene from δ 151.051 to 160.723 ppm. The number of signals and peaks in the ^13^C NMR spectrum of compound (**5**) (Fig. 7) corresponds to the number of carbons in the compound. The shifts of the C1, C8, C7, and C14 peaks suggest that these atoms were involved in the coordination (Table 3). The signals corresponding to the δ(C4-OH) proton and δ(CH = N) carbon (in pyridine) groups are unchanged in the ^1^H and ^13^C NMR spectra of the complex, indicating that these groups do not take part in complexation.

### Mass spectra

**Fig. 8:**
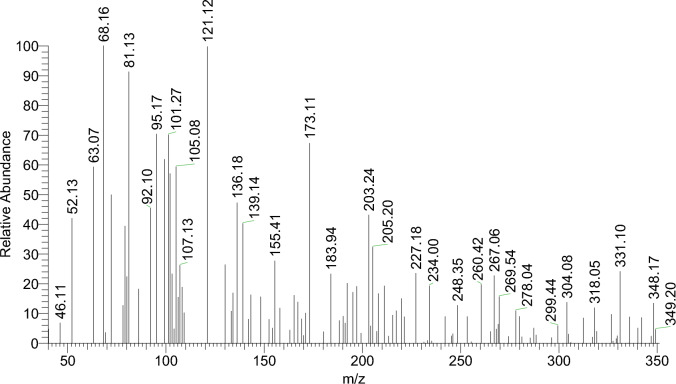
The mass spectrum of the ligand (H_2_L).

**Scheme 3:**
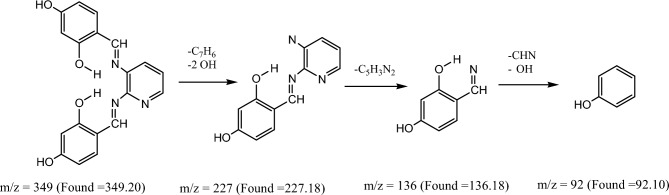
The fragmentation of the mass spectrum of the ligand (H_2_L).

**Scheme 4:**
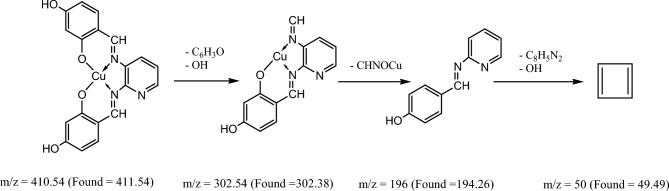
The fragmentation of the mass spectrum of Cu(II) **(1)**.

**Scheme 5:**
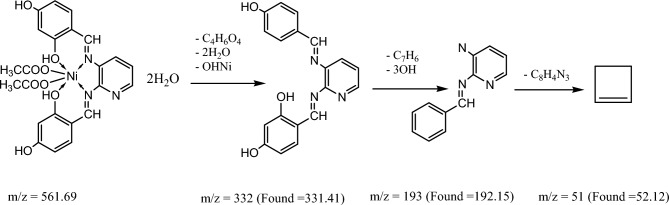
The fragmentation of the mass spectrum of Ni(II) complex **(4)**.

**Scheme 6:**
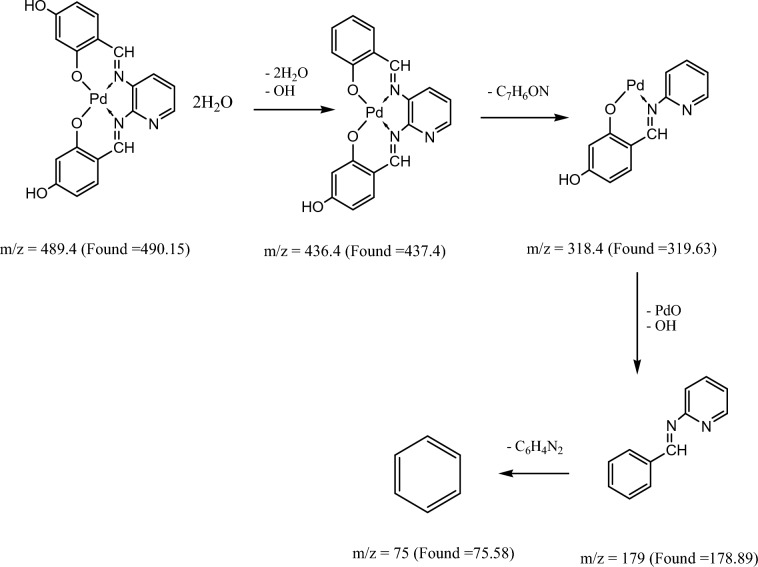
The fragmentation of the mass spectrum of Pd(II) complex **(5)**.

Figure 8 and Figures S1–S3 display the mass spectra of the ligand (H_2_L) and its complexes (**1**, **4,** and **5**) as evidence that the empirical formula corresponds to the molecular ion peaks. A molecular ion peak forms at m/z = 349 amu according to the ligand empirical formula, [C_19_H_15_N_3_O_4_]. This ion peak separated into separate stable peaks at m/z = 227, 136, and 92 amu, which represent the losses of 2OH + C_7_H_6_, C_5_H_3_N_2_, and CH_2_NO, respectively, as shown in Scheme 3. Complex **(1)** exhibited a molecular ion peak with m/z = 410.54 amu [C_19_H_13_N_3_O_4_Cu]. This peak then broke down into distinct stable ion peaks with m/z = 302.54, 196, and 50 amu (Scheme 4), which correspond to the losses of OH + C_6_H_3_O, CHNOCu, and OH + C_8_H_5_N_2_, respectively. The structure of Ni(II) complex **(4)** is consistent with this molecular ion peak at m/z = 561.69 amu [C_23_H_21_N_3_O_4_Ni] 2H_2_O. As can be seen in Scheme 5 and Figure S3, it loses 2H_2_O + C_4_H_7_O_5_Ni, C_7_H_9_O_3,_ and C_8_H_4_N_3_ with m/z = 332, 193, and 51 amu, respectively, as shown in Scheme 6 and Figure S2. The Pd(II) complex **(5)** displays a molecular ion peak at m/z = 489.4 amu that is consistent with the structure of [C_19_H_13_N_3_O_4_Pd] 2H_2_O, losing 2H_2_O + OH, C_7_H_6_NO, OH + PdO, and C_6_H_4_N_2_ with m/z = 436.4, 318.4, 179, and 75 amu, respectively.

### Magnetic susceptibility result

The magnetic susceptibility measurements contributed to the determination of complexes structure. These measurements provide information about the type of bonding and the strength of the ligand field of complexes, and also give information about the number of unpaired electrons. Octahedral Co(II) complex, however, exhibits 4.82 B.M. show paramagnetic behavior with three unpaired electrons, indicating a high-spin octahedral configuration. The magnetic moment of Cu(II) complex was seen at 1.82 B.M., corresponding to one unpaired electron, and shows the absence of spin–spin interactions. The magnetic moment of the Ni(II) complex was seen within the range of 3.08 B.M for the octahedral Ni(II) complex. The Mn(II) complex shows 5.09 B.M. at room temperature, corresponding to five unpaired electrons, which suggests octahedral geometry^40,44^.

### Electronic spectra

The absorption band of free Schiff base appeared at 29,675 cm^-1^ is attributed to the azomethine chromophore π → π* transitions, and the bands at higher energies 36,260 and 32,460 cm^-1^ are associated with π → π* transitions of aromatic benzene ring.

The electronic spectrum of the present copper(II) complex (**1**) showed absorption band at geometry 21,186 cm^−1^ assigned to ^2^B_1g_ → ^2^A_1g_ transition, which corresponds to a square planar geometry around Cu(II) ion^56–58^. The absence of absorption below 10,000 cm^−1^ excludes the possibility of a tetrahedral geometry for the complexes. An additional band at 25,935 cm^−1^ attributed to INCT may also be present in the spectra of square planar complexes^56–58^. The magnetic moment of the Cu(II) complex is 1.79 BM, which represents 3d^9^ paramagnetic Cu(II) complexes and is also supportive of square planar. Magnetic susceptibility measurement showed that this complex is paramagnetic, which corresponds to the + 2 oxidation state of Cu(II) complex.

The Co(II) complex (**2**) exhibited two bands at 20,620 [^4^T_1g_ → ^4^A_2g_ (F)] and16048 cm^-1^ [^4^T_1g_ → ^4^T_1_g (P)] transitions, due to d-d transitions and separately, which is the specificity of the octahedral environment around the Co(II) ion^59^. The magnetic moment value of the complex is 4.82 B.M.The electronic field parameters Dq(590), B(952), β(0.81), and υ_2_/υ_1_(2.03_)_ support the octahedral environment of the complex (**2**). The values of the Racah parameter are less than those of free cobalt ions, suggesting electron delocalization and spin orbital overlap. Nephelauxetic ratio values smaller than one suggest the covalent nature of the metal-Schiff base connection^37,49^. Because of orbital overlaps and significant electron transit across the Co(II) ion, the value of B (interionic repulsion factor) is less than 971         cm^-1^^60^,^61^.The room temperature magnetic moment of the manganese (II) complex **(3)** lies at 5.01 B.M., which is lower than expected for a high-spin 3d^5^ system. This may be due to super exchange via overlap of the metal orbital’s with the orbital’s of the coordinated oxygen atoms. The electronic spectrum of this complex exhibits three bands at 11,050. 15,380 and 18,520 cm^-1^ attributable to (^6^A_1_ →^4^T_1g_) (G), (^6^A_1_→^4^T_2g_) (G), and (^6^A_1_→^4^E_1g_) (G), respectively suggesting a octahedral coordinated manganes(II)^56^.

Two of the three bands expected for the octahedral Ni(II) complex **(4)** are pointed at 23,201 and 20,988 cm^−1^ in the Ni(II) complex spectrum. The absence of the third transition band is probably due to the low intensity. These two values are attributed, respectively, to ^3^A_2g_ →^3^T_2g_ (P) and ^3^A_2g_ →^3^T_1g_ (F) transitions for the octahedral environment in Ni(II) complex. The magnetic moment of 3.08 B.M. lies within the range reported for octahedral Ni(II) complexes^62,63^.

The electronic spectrum of Pd(II) complex **(5)** exhibits three bands at 25,310 cm^-1^ [^1^A_1g_ → ^1^E_g_], 19,525 cm^-1^ [^1^A_1g_ → ^1^B_1g_] and 14,830 cm^-1^ [^1^A_1g_ → ^1^A_2g_] transition. This complex has been proposed to possess diamagnetic and square planar geometry^56^.

### The analysis of X-ray diffraction

Single crystals of the ligand (H_2_L) and its metal complexes could not be prepared for X-ray diffraction (XRD), and hence, the powder diffraction data were obtained for structural characterization. Structure determination by X-ray powder diffraction data has gone through a recent surge since it has become important to get to the structural information of materials that do not yield good-quality single crystals. X-ray diffraction analysis was utilized to elucidate the structure of the ligand or complexes.

**Fig. 9:**
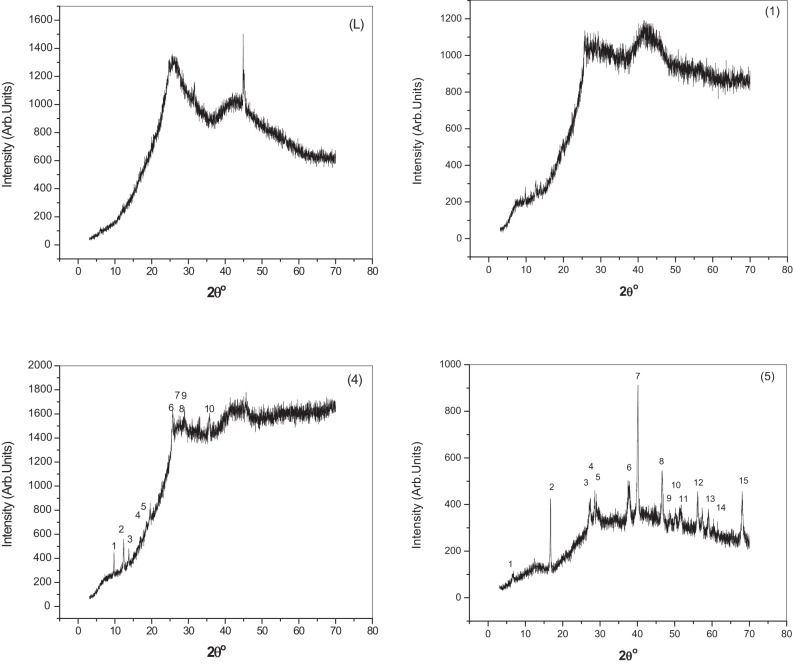
X-ray diffraction patterns of the ligand (H_2_L) and metal complexes **(1), (4),** and **(5)**.

The X-ray diffraction analysis of the ligand (H_2_L) and its complexes** (1)**, **(4)**, and **(5)** is shown in Fig. 9. The ligand and its complex **(4)** display a wide peak that indicates the amorphous phases, while the complex **(1)** and complex **(5)** exhibit multiple diffraction peaks that indicate the polycrystalline phases^23,64^. Tables 4 and 5 display the values of the estimated lattice parameters (α, β, and γ), inter-planar spacing (d), Miller indices (hkl), and errors for lattice constants of complex **(1)** and complex **(5)** as obtained using the CHEKCELL software^25^.

**Table 4:** Crystallographic data for Cu(II) complex **(1)**.

Sys*. monoclinic S.G*. p21 a = 14.5348Å b = 3.7479Å c = 10.2359Å α = 90.00º β = 101.03º γ = 90.00 º					
Peak no	2θ_Obs._ (º)	d_Obs._ (Å)	2θ_Calc._ (º)	d_Calc._ (Å)	*(hkl*)
1	9.7547	9.06	9.7421	9.0716	$\overline{1} 01$
2	12.3741	7.1473	12.3986	7.1332	$200$
3	13.7946	6.4143	13.7711	6.4254	$\overline{2} 01$
4	18.6556	4.7525	18.6438	4.7555	$300$
5	19.5785	4.5311	19.5555	4.5358	$\overline{2} 02$
6	25.7451	3.4576	25.6975	3.4639	$\overline{1} 11$
7	26.6639	3.3405	26.5953	3.3498	$003$
8	27.1100	3.2865	27.1433	3.2826	$\overline{2} 03$
9	27.4791	3.2432	27.5298	3.2374	$\overline{2} 11$
10	35.6757	2.5147	35.7185	2.5117	$004$

Sys.*: System.

S. G*.: Space group.

**Table 5:** Crystallographic data for Pd(II) complex **(5)**.

Sys*. monoclinic S.G*. C2 a = 20.2182Å b = 2.5848Å c = 13.4112Å α = 90.00º β = 95.86 º γ = 90.00 º					
Peak no	2θ_Obs._ (º)	d_Obs._ (Å)	2θ_Calc._ (º)	d_Calc._ (Å)	*(hkl*)
1	6.6300	13.3211	6.6200	13.3412	$001$
2	16.7148	5.2997	16.6676	5.3146	$202$
3	27.3156	3.2623	27.2762	3.2669	$\overline{2} 04$
4	28.5552	3.1234	28.5320	3.1259	$\overline{6} 02$
5	29.0240	3.074	29.0301	3.0734	$204$
6	37.7510	2.381	37.7240	2.3827	$112$
7	40.1088	2.2463	40.1919	2.2419	$312$
8	46.6422	1.9458	46.6183	1.9467	$\overline{6} 06$
9	48.7699	1.8657	48.6690	1.8658	$\overline{5} 14$
10	50.1922	1.8162	50.2589	1.8139	$712$
11	51.5793	1.7705	51.5476	1.7715	$606$
12	56.1782	1.636	56.2735	1.6335	$\overline{4} 08$
13	57.3682	1.6049	57.3370	1.6057	$316$
14	59.0440	1.5632	59.0559	1.563	$\overline{12} 04$
15	68.0964	1.3758	68.0956	1.37582	$1402$

Sys.*: System.

S. G*.: Space group.

The average crystallite size (ξ) was determined from the XRD patterns using the Debye–Scherrer equation^23,38^:

$$\, \xi = \frac{{0.95_{{}} \lambda }}{{\beta_{1/2} \cos \theta }}$$

where the wavelength is λ = 1.540598Å, the incidence angle is (θ), and the breadth of the reference diffraction peak, measured in radians at half maximum, is β_1/2_. The value of ξ is used to determine the dislocation density’s δ value^38^:

$${\updelta } = \frac{1}{{\xi^{{2}} }}$$

For complex **(1)** and complex **(5)**, the ξ is located at 51 and 35 nm, respectively. For complexes** (1)** and **(5)**, the δ values are 3.84 × 10^−4^ and 8.16 × 10^−4^ nm^−2^, respectively. This suggests that complex **(5)** is a more polycrystalline phase than complex **(1)**.

### Thermogravimetric(TGA) analysis

**Fig. 10:**
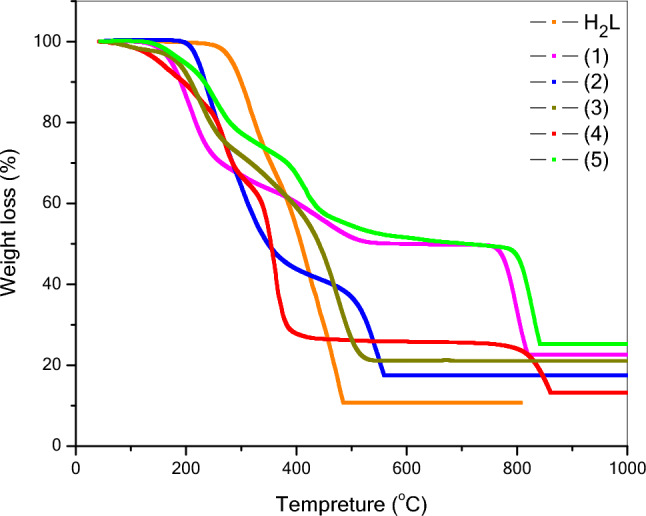
The TGA curves of the ligand (H_2_L) and its metal complexes **(1–5)**.

Figure 10 displays the thermogravimetric analysis data (TGA) of the Schiff base ligand and its complexes, while Table 6 provides a summary of the mass percentage losses. The TGA data show two phases of breakdown for the Schiff ligand (H_2_L). The first phase is linked to the loss of C_7_H_7_O_2_ and happens between 50 and 370 °C. The second stage is the primary component of the ligand (C_9_H_8_N_3_O_2_) breaking down between 370 and 525 °C, leaving behind three carbon atoms as a residue.

**Table 6:** TGA thermal data of theligand(H_2_L) and its metal complexes**(1–5)**.

Compound	Temperature range (°C)	TG weight loss %		Assignments
		Found	Calc	
**H**_**2**_**L**	50–370	35.41	35.24	C_7_H_7_O_2_
	370–525	54.04	54.44	C_9_H_8_N_3_O_2_
	Remain	10.55	10.32	3C
**(1)**	50–242	26.87	26.79	C_6_H_6_O_2_
	242–516	22.65	22.65	C_5_H_3_NO
	516–893	27.94	28.26	C_7_H_4_N_2_
	Remain	22.54	22.3	CuO + C
**(2)**	50–367	52.28	51.61	C_12_H_14_O_6_ + 2H_2_O
	367–648	30.31	30.79	C_9_H_7_N_3_O
	Remain	17.41	17.61	CoO + 2C
**(3)**	50–256	19.29	19.3	3H_2_O + 2OH
	256–412	22.75	22.37	C_8_H_6_
	412–615	37.13	37. 5	C_9_H_5_N_3_O
	Remain	20.83	20.82	MnO + 2C
**(4)**	50–303	33.51	33.47	2H_2_O + C_4_H_8_O_6_
	303–386	37.11	36.68	C_14_H_8_NO
	386–917	16.83	16.56	C_5_H_5_N_2_
	Remain	12.98	13.29	NiO
**(5)**	50–277	19	19.41	2H_2_O + 2OH + C_2_H
	277–454	23.69	23.91	C_7_H_3_NO
	454–870	32.01	31.67	C_10_H_7_N_2_
	Remain	25. 3	25.01	PdO

^a^Numbers as given in Scheme 2.

The Cu(II) complex (**1**) showed three stages of decomposition in its TGA curve. The loss of C_6_H_6_O_2_ is the first step at temperatures ranging from 50–242 °C and the second step at temperatures ranging from 242–516 °C due to the loss of C_5_H_3_NO. In the breakdown of C_7_H_4_N_2_ in the third stage at temperatures ranging from516 to 893 °C, the residue is CuO and one carbon atom. There is incomplete decomposition, and the residue is CuO + C.

The Co(II) complex **(2)** shows two stages of decomposition in the TGA curve. The loss of C_12_H_14_O_6_ + 2H_2_O is the cause of the first step, and at temperatures ranging from 50 to 367 °C. Due to the loss of C_9_H_7_N_3_O in the second step at temperatures ranging from367-648 °C, the residue is CoO and two carbon atoms.

The Mn(II) complex **(3)** shows three stages of decomposition in the TGA curve. The loss of 3H_2_O + 2OH is the cause of the first step, and at temperatures ranging from 50 to 256 °C. Due to the loss of C_8_H_6_ in the second step at temperatures ranging from 256 to 412 °C, and the breakdown of C_9_H_5_N_3_O in the third stage at temperatures ranging from 412 to 615 °C, the residue is MnO and two carbon atoms due to incomplete decomposition.

Ni(II) complex (**4**) shows three stages of decomposition in the TGA curve. The first stage, due to the loss of 2H_2_O + C_4_H_8_O_6_, happens at 50–303 °C. The second stage is due to the loss of C_14_H_8_NO breaking down at 303–386 °C. The third stage is due to the loss of C_5_H_5_N_2_ breaking down at 386–917 °C. The residue is NiO.

For Pd(II) complex (**5**) shows three stages of decomposition in the TGA curve and the loss of 2H_2_O + 2OH + C_2_H in the first step at temperatures ranging from 50–277 °C. In the second step, loss of C_7_H_3_NO at temperatures ranging from 277 to 454 °C. The third stage is due to the loss of C_10_H_7_N_2_ breaking down at 454–870 °C. The residue is PdO.

### Calf thymus DNA binding study

**Fig. 11:**
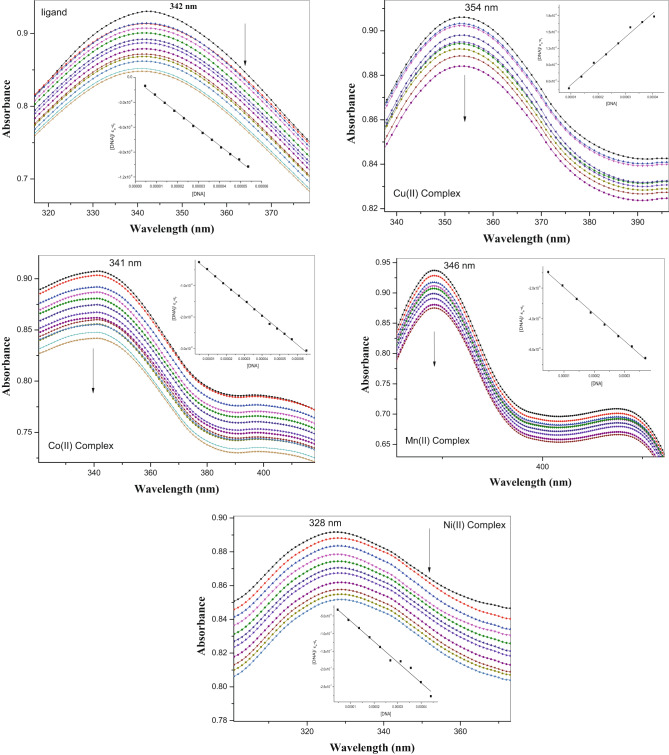
Absorption spectrum of the ligand (H_2_L) and metal complexes **(1–5)** in buffer pH 7.2 in the presence of increasing amounts of CT-DNA. Arrows indicate the changes in absorbance which decrease by increases the CT-DNA concentration. Inside the plot of [DNA]/(ε_a_ − ε_f_) as a function of DNA concentration as determined from the absorption spectral data of ligand (H_2_L) and metal complexes**(1–5)** and calf thymus DNA.

Using electronic absorption spectroscopy, the intercalation of the ligand (H_2_L) and complexes **(1–5)** with calf thymus DNA, the intrinsic binding constant (K_b_) of the calf thymus DNA was ascertained. The calf thymus DNA assays were conducted at room temperature using fixed ligand concentrations and a progressive increase in the calf thymus DNA (CT-DNA) concentration. The absorption spectra acquired by tracking the absorption intensity of the charge transfer spectral bands 342, 354, 341, 346, 328, and 336 nm for the ligand (H_2_L) and complexes **(1–5)**, respectively, were used to calculate the K_b_ to CT-DNA. As the concentration of CT-DNA increased, the absorption spectra of the ligand and complexes (**1**–**5**) were seen to have a hypochromic effect and to decrease with a little red shift (~ 1–2 nm) (Fig. 11). The absorption spectral technique was used to calculate the intrinsic binding constants (K_b_) values of the ligand (H_2_L) and its complexes (**1**–**5**) with CT-DNA. The intercalation of ligand and complexes (**1**–**5**) with Calf thymus DNA displays hypochromism because the intercalation mechanism involves a strong stacking contact between an aromatic chromophore and the base pairs of DNA.

The results showed that the intrinsic binding constants (K_b_) values in the range 4.56 × 10^5^—15.26 × 10^5^ M^-1^ for the ligand and its metal complexes, as listed in Table 7.

**Table 7:** The intrinsic binding constants (K_b_) values of the ligand and its metal complexes with calf thymus DNA.

Compound	Intrinsic binding constants (K_b_) (M^-1^)	Lamda (nm)
Ligand	6.93 × 10^5^	342
Cu(II) complex	4.56 × 10^5^	354
Co(II) complex	7.64 × 10^5^	341
Mn(II) complex	8.37 × 10^5^	346
Ni(II) complex	11.28 × 10^5^	328
Pd(II) complex	15.26 × 10^5^	336

These results offer compelling evidence that complexes with the DNA helix can attach more readily when the terminal nitrogen is substituted with a Schiff base ligand that has a greater capacity to donate electrons. It is clear from the results of the electronic absorption experiments that all of the complexes have positive interactions with CT-DNA. Furthermore, compared to the other complexes, the Pd complex was shown to have a higher percentage of hypocromism and stronger binding constant values, suggesting a higher affinity for DNA. A comparative summary has been displayed in Table 8 to compare the DNA binding ability of the reported complexes with those of the previously reported similar compounds^65–73^.

**Table 8:** DNA binding constants, electronic absorption spectral features, and comparative literature values for the ligand and its metal(II) complexes.

Metal complex	DNA binding constant (K_b_) × 10^5^ (M⁻^1^)	Binding mode	The reported literature range
H₂L	4.56	Minor hypochromism, Δλ ≈ 1 nm → weak intercalation	Free Schiff ligands typically show K_b_ 10^4^–10^5^; extended aromatic ligands may reach upper 10^5^^65–67^
Mn(II) complex	8.73	Moderate hypochromism (8%), intercalative	Mn-Schiff complexes show a broad range (10^4^–10^5^),^68,69^
Co(II) complex	9.84	Bathochromic shift 2 nm; intercalative + electrostatic	Co-Schiff complexes frequently fall in the 10^5^–10⁶ region, Matches Co(II)–Schiff base data (K_b_ = 8.2–10.6 × 10^5^)^66,70,71^
Ni(II) complex	12.35	Hypochromism 10%, red shift ≈ 2 nm	Ni-Schiff complexes report K_b_in the 10^5^–10⁶ range; examples include 1.3 × 10^5^ depending on ligand planarity^69^
Cu(II) complex	10.42	Strong bathochromism (3 nm), π–π stacking	Cu-Schiff complexes show K_b_from 10^4^ up to 10⁶; planar Cu complexes reach 7 × 10^5^^72^
Pd(II) complex	15.26	Pronounced hypochromism (12%), Δλ = 3 nm → strong intercalation	Pd(II)–Schiff complexes reported K_b_values ~ 4.24 × 10^5^ and 4.98 × 10^5^ in a recent Pd–Schiff study^73^

### Molecular docking

Numerous possible adduct structures are produced by molecular docking and are subsequently examined and categorized by means of the software’s scoring system. The ligand binding energy, free energy, and stability may all be found out by the docking method^6,54,74,75^. Molecular docking creates many potential adduct structures, which are then analyzed and classified using the software’s scoring algorithm. The docking approach can provide information on the ligand binding energy, free energy, and stability.

Molecular docking studies have been applied to ligand and Cu(II), Co(II), Ni(II), Mn(II), and Pd(II) complexes (**1–5**) against the breast cancer MCF-7 cell line (PDB IDs 1QNT, 3FC2, and 4AJY) as additional evidence for biological screening investigations. The selection of the target proteins 1QNT, 3FC2, and 4AJY for this molecular docking study was guided by their established roles in breast cancer progression and their potential as therapeutic targets.

The results are shown in Table 9, revealing a solid match between docking and experimental data. Our docking models demonstrated that the inhibitor molecules will engage successfully with certain proteins associated with their active sites. All of the compounds seemed to have acceptable RMSD values.

**Table 9:** Docking scores and energies of the ligand (H_2_L) and its metal complexes**(1–5)** with breast cancer MCF-7 cell line PDB IDs: 1QNT, 3FC2, and 4AJY) receptors.

Protein	Compounds	S	rmsd_refine	E_conf	E_place	E_score1	E_refine	E_score2
1QNT	H_2_L ligand	− 5.95868	0.659173	10.68625	− 68.3226	− 10.6732	− 33.0942	− 5.95868
	Cu(II) complex	− 5.68633	1.069136	51.54837	− 81.2294	− 8.86551	− 30.3419	− 5.68633
	Co(II) complex	− 6.55711	3.709362	− 609.603	− 76.4144	–	− 34.6482	− 6.55711
	Mn(II) complex	− 6.23818	2.058545	− 669.668	− 81.1356	− 11.2171	− 38.4602	− 6.23818
	Ni(II) complex	− 5.50322	3.753933	− 715.289	− 84.9715	–	− 26.5888	− 5.50322
	Pd(II) complex	− 5.6987	0.549175	− 18.8123	− 64.6971	− 9.36652	− 29.8227	− 5.6987
3FC2	H_2_L ligand	− 6.8478	0.949552	20.45212	− 95.0352	− 12.2369	− 33.0725	− 6.8478
	Cu(II) complex	− 6.29598	0.750893	55.12846	− 84.773	− 10.141	− 32.1531	− 6.29598
	Co(II) complex	− 7.28522	1.957767	− 605.339	− 114.352	–	− 33.9141	− 7.28522
	Mn(II) complex	− 5.77981	4.858589	− 700.562	− 93.6065	− 15.5317	− 33.7155	− 5.77981
	Ni(II) complex	− 5.70019	3.504526	− 717.292	− 118.552	–	− 22.7389	− 5.70019
	Pd(II) complex	− 6.16648	3.032492	− 20.4085	− 90.8586	− 12.3593	− 32.8833	− 6.16648
4AJY	H_2_L ligand	− 5.70982	1.071604	3.339897	− 84.5857	− 10.9893	− 29.9898	− 5.70982
	Cu(II) complex	− 4.95635	1.209751	51.51311	− 73.5334	− 8.55917	− 25.1877	− 4.95635
	Co(II) complex	− 5.79769	3.045317	− 607.982	− 81.1983	–	− 29.3464	− 5.79769
	Mn(II) complex	− 6.1004	3.41909	− 702.272	− 68.0269	− 10.5798	− 37.0389	− 6.1004
	Ni(II) complex	− 5.54071	2.165108	− 712.464	− 83.2707	–	− 26.8393	− 5.54071
	Pd(II) complex	− 5.40537	1.850313	− 20.5825	− 55.9936	− 12.8776	− 27.2834	− 5.40537

Co(II) complex exhibited effective in silico inhibition of (1QNT) and (3FC2) proteins based on low docking scores (-6.55711 and -7.28522 kcal/mol ), respectively. Co-complex is bound with (1QNT) through amino acid residues (GLU 77 and LYS 104) via O 21 and 6-ring, respectively. while interacting with (3FC2) through the amino acid residue LEU 59 via the 6-ring.

On the other side, the interaction of (4AJY) with tested compounds showed that the Mn(II) complex is a more effective inhibitor with a binding energy of (-6.1004kcal/mol) and binding interactions with the residue ASP 47.

**Fig. 12:**
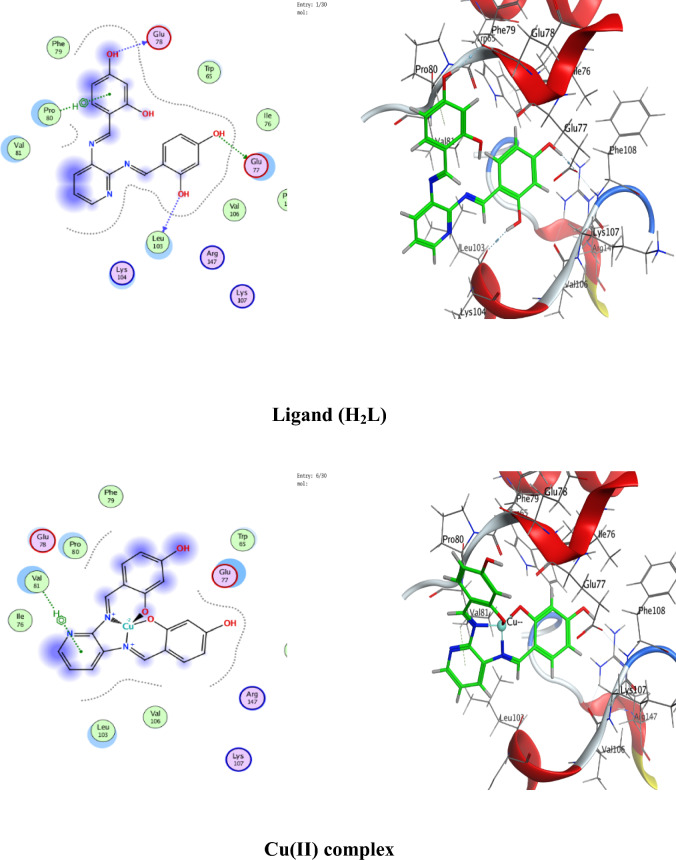

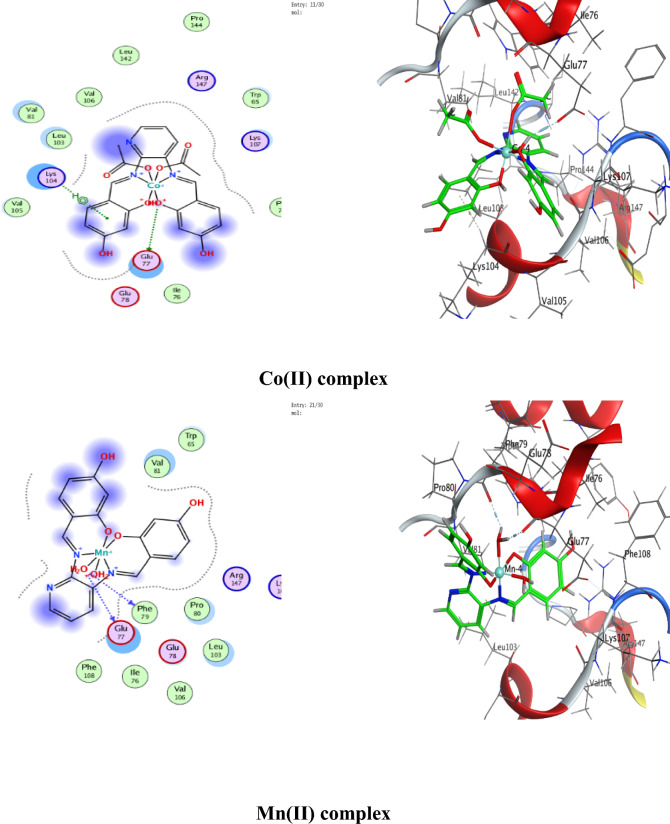

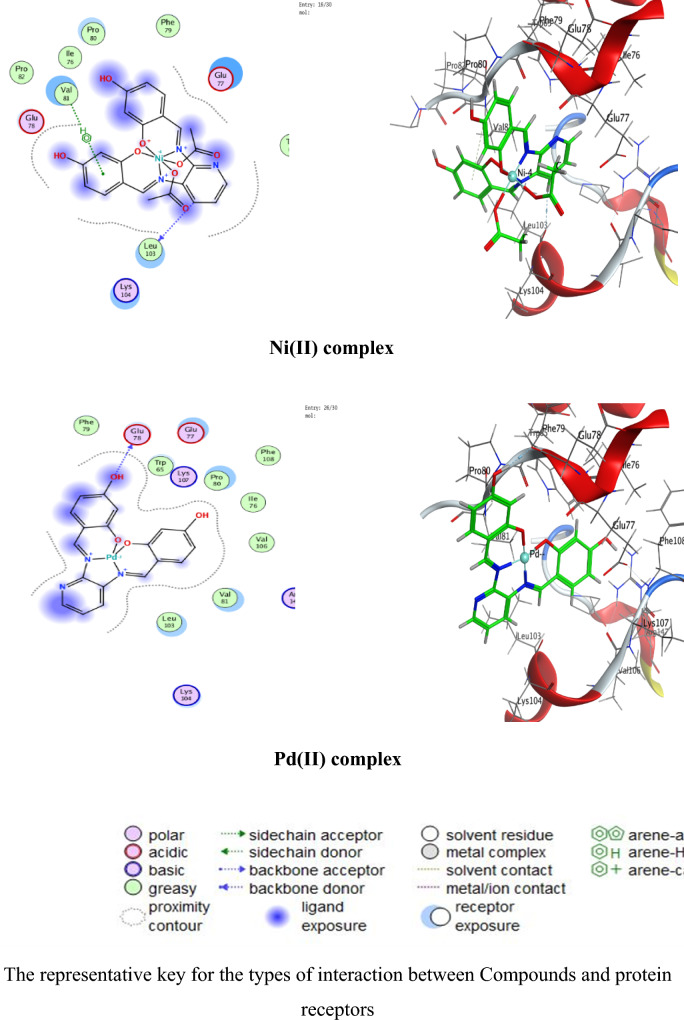
2D and 3D diagrams show the interaction between the ligand (H_2_L) and metal complexes (**1–5**) with the active sites of the 1QNT protein.

**Fig. 13:**
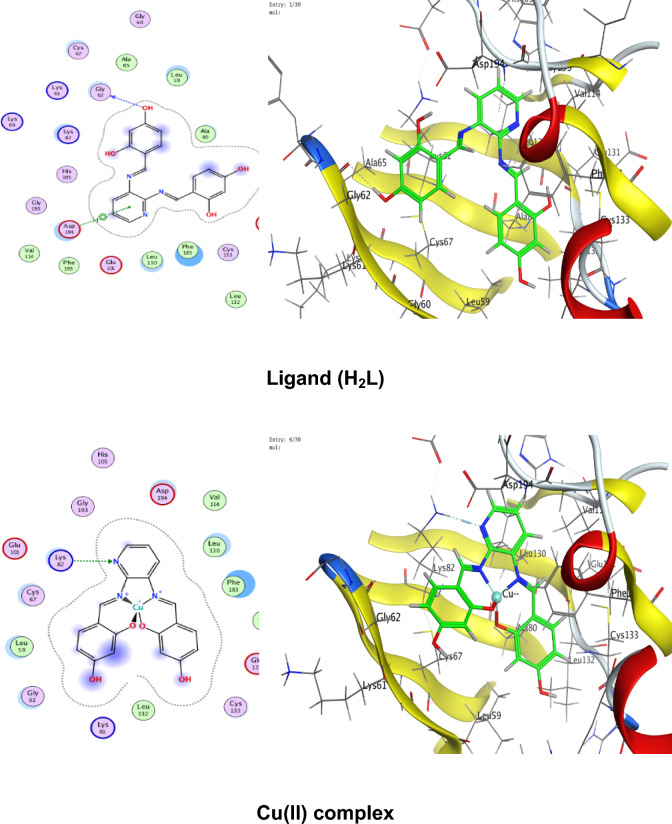

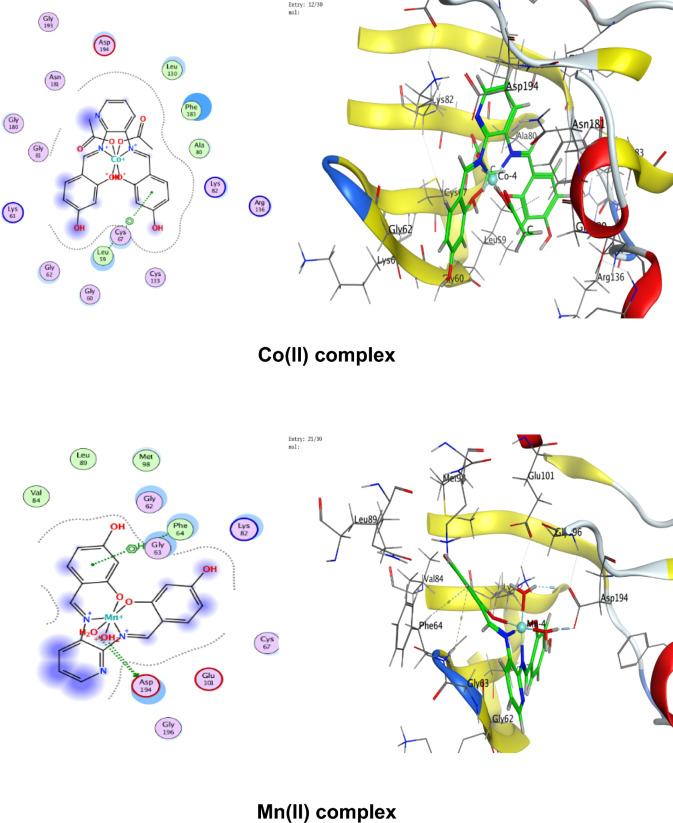

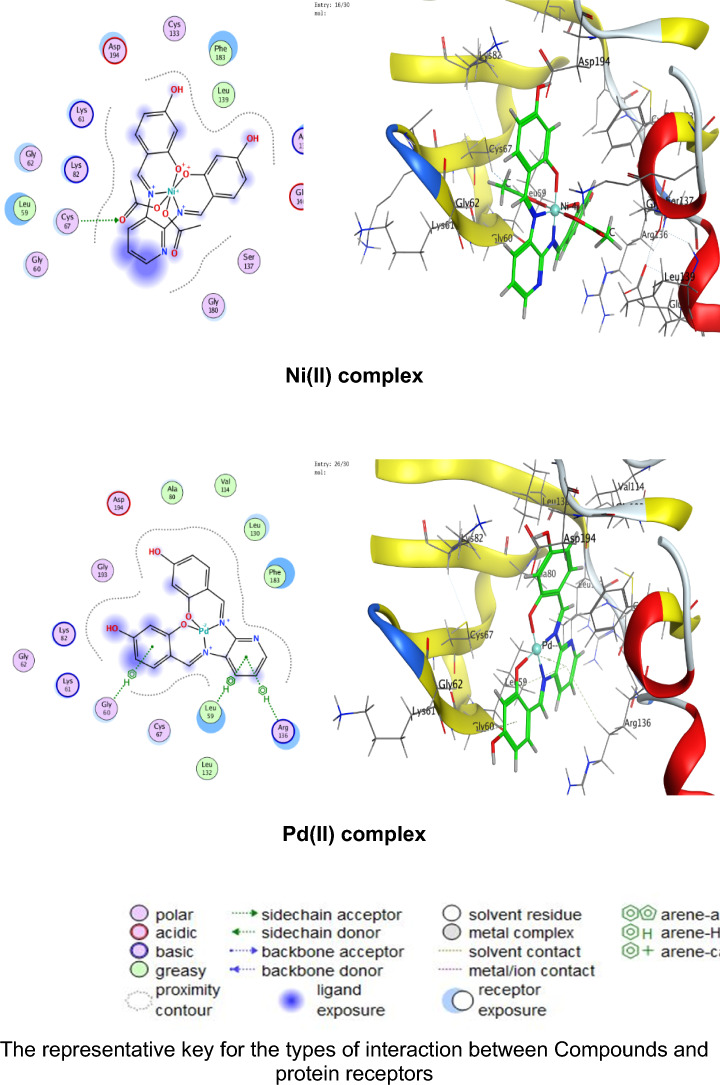
2D and 3D diagrams show the interaction between the ligand (H_2_L) and metal complexes (**1–5**) with the active sites of 3FC2 protein.

**Fig. 14:**
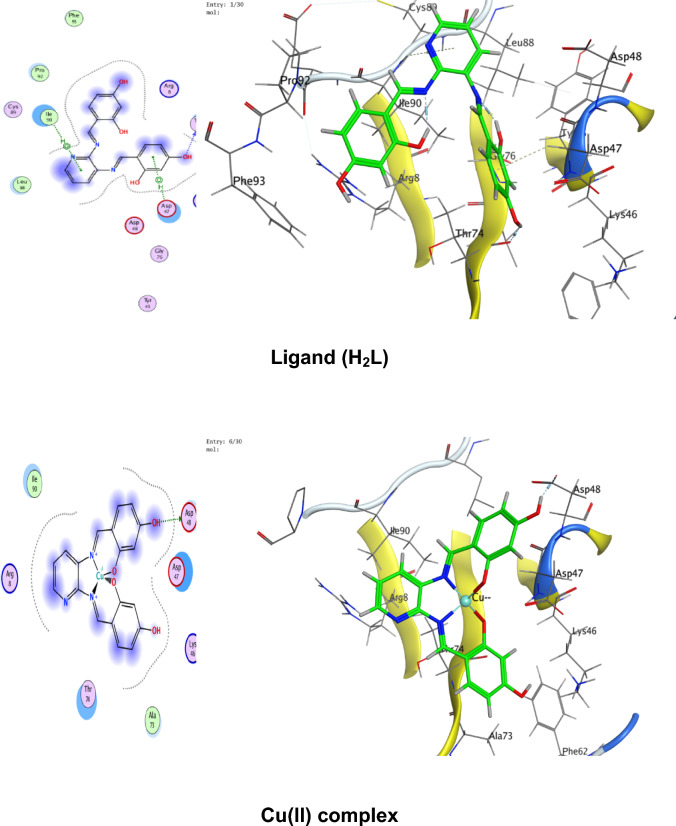

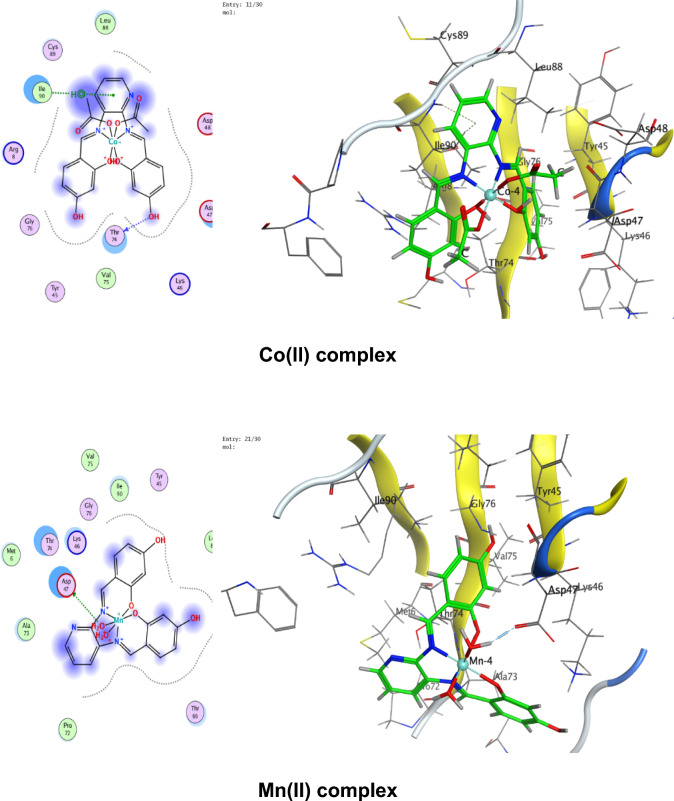

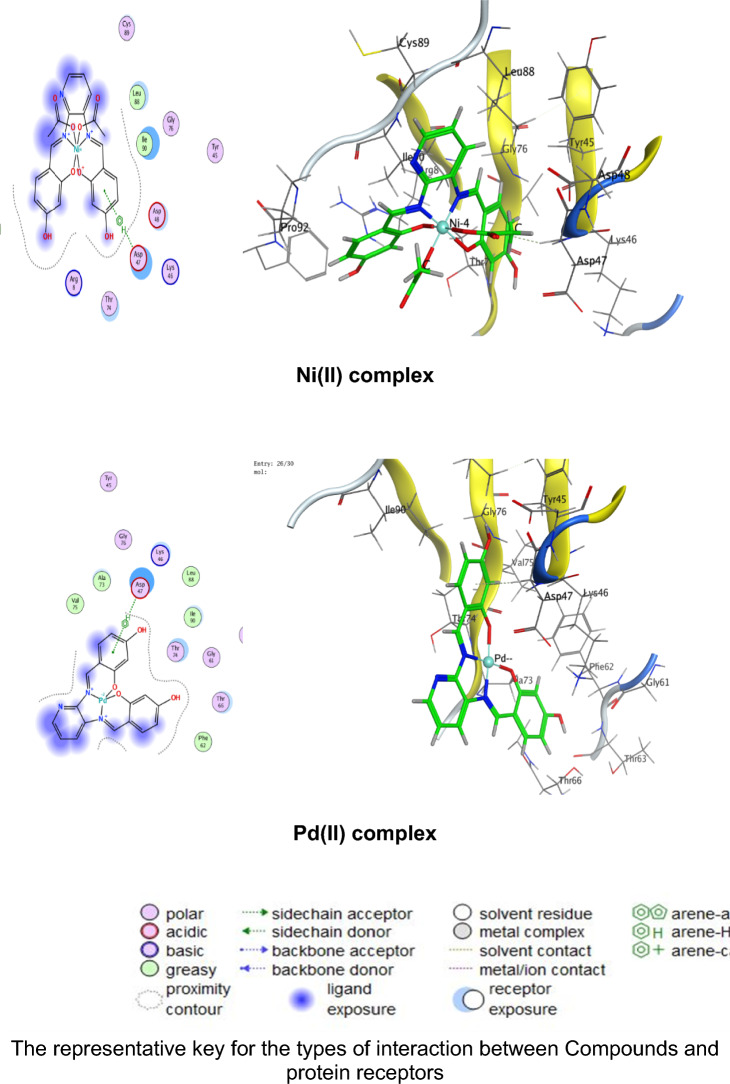
2D and 3D diagrams show the interaction between the ligand (H_2_L) and metal complexes (**1–5**) with the active sites of the 4AJY protein.

Table 10 lists the hydrogen bonds formed by the ligand and Cu(II), Co(II), Ni(II), Mn(II), and Pd(II) complexes(**1–5**) with the proteins under investigation. The best-fitted 2D and 3D poses chosen by the examined compounds are represented in Figs. 12, 13 and 14.

**Table 10:** Interaction between the ligand (H_2_L) and its metal complexes** (1–5)** with the breast cancer MCF-7 cell line PDB IDs: 1QNT, 3FC2, and 4AJYreceptors.

Protein	Compounds	Ligand	Receptor	Interaction	Distance	E (kcal/mol)
1QNT	H_2_L ligand	O 19	O GLU 78 (A)	H-donor	3.01	− 1.4
		O 29	O LEU 103 (A)	H-donor	3.06	− 2.0
		O 31	OE2 GLU 77 (A)	H-donor	2.91	− 1.1
		6-ring	CA PRO 80 (A)	pi-H	3.55	− 0.5
	Cu(II) complex	6-ring	CB VAL 81 (A)	pi-H	3.89	− 0.6
	Co(II) complex	O 21	OE1 GLU 77 (A)	H-donor	2.92	− 5.8
		6-ring	CA LYS 104 (A)	pi-H	4.01	− 0.7
	Mn(II) complex	O 44	O GLU 77 (A)	H-donor	2.63	− 1.6
		O 44	O PHE 79 (A)	H-donor	2.63	− 1.9
	Ni(II) complex	C 17	O LEU 103 (A)	H-donor	3.55	− 0.6
		6-ring	N VAL 81 (A)	pi-H	4.53	− 0.5
	Pd(II) complex	O 29	O GLU 78 (A)	H-donor	3.07	− 1.7
3FC2	H_2_L ligand	O 19	O GLY 62 (A)	H-donor	3.21	− 0.5
		6-ring	N ASP 194 (A)	pi-H	3.25	− 0.6
	Cu(II) complex	N 12	NZ LYS 82 (A)	H-acceptor	3.05	− 6.6
	Co(II) complex	6-ring	CD1 LEU 59 (A)	pi-H	4.56	− 0.6
	Mn(II) complex	O 41	OD2 ASP 194 (A)	H-donor	2.58	− 5.0
		O 44	OD1 ASP 194 (A)	H-donor	3.17	− 1.9
		6-ring	N PHE 64 (A)	pi-H	4.54	− 1.1
		6-ring	CB PHE 64 (A)	pi-H	4.36	− 0.6
	Ni(II) complex	O 46	CB CYS 67 (A)	H-acceptor	3.12	− 0.5
	Pd(II) complex	6-ring	CB LEU 59 (A)	pi-H	4.47	− 0.5
		6-ring	CD1 LEU 59 (A)	pi-H	3.77	− 1.4
		6-ring	CA GLY 60 (A)	pi-H	3.77	− 1.1
		6-ring	CG ARG 136 (A)	pi-H	4.76	− 0.5
4AJY	H_2_L ligand	O 19	O THR 74 (B)	H-donor	2.80	− 2.2
		6-ring	CA ASP 47 (B)	pi-H	3.69	− 0.5
		6-ring	N ILE 90 (B)	pi-H	4.27	− 2.6
	Cu(II) complex	O 19	OD2 ASP 48 (B)	H-donor	2.89	− 2.3
	Co(II) complex	O 31	O THR 74 (B)	H-donor	2.83	− 2.2
		6-ring	N ILE 90 (B)	pi-H	3.86	− 1.2
		6-ring	CB ILE 90 (B)	pi-H	4.30	− 0.5
	Mn(II) complex	O 44	OD1 ASP 47 (B)	H-donor	2.58	− 5.1
	Ni(II) complex	6-ring	CA ASP 47 (B)	pi-H	3.58	− 0.5
	Pd(II) complex	6-ring	CA ASP 47 (B)	pi-H	3.51	− 0.6

**1QNT** and **3FC2**: Both are implicated in pro-survival signaling. The strong binding of Co(II) complex to these proteins (Co(II) complex with 1QNT: S = -6.55711; with 3FC2: S = -7.28522) suggests potential disruption of its enzymatic or receptor functions, thereby halting tumor progression.

**4AJY**: Its role in DNA repair makes it a strategic target for combination therapies. The moderate affinity of ligand and its complexes (e.g., G with 4AJY: S = -5.70982) could impair DNA repair efficiency, rendering cancer cells vulnerable to secondary treatments.

- Targeting 1QNT and 3FC2 aligns with strategies to inhibit proliferation and metastasis, while 4AJY inhibition could enhance the efficacy of DNA-targeted therapies.
- The superior performance of Co(II) and Mn(II) complexes against these proteins highlights their potential as dual-targeting agents, capable of blocking multiple on cogenic pathways simultaneously.

In vitro inhibitory actions are characterized by enhanced interaction between compounds and receptors.

### Antimicrobial properties

#### Agar well diffusion test

The antimicrobial activity of ampicillin, a common antibacterial, and terconazole, a common antifungal, as well as 2,3-diaminopyridine, 2,4-dihydroxy benzaldehyde, H_2_L ligand, and metal complexes, was investigated using the agar well diffusion test. Microbes included in this study included fungi *A. flavus*, *A. solani*, and *C. albicans*, bacteria *B. cereus*, *S. epidermidis*, *E. faecalis,* and *Salmonella* sp.^76–79^. The microbicidal results of the produced compounds are shown in Tables 11 and 12. The results can be explained as follows when interpreted:

1. The 2,4-dihydroxy benzaldehyde showed the highest antibacterial activity in comparison with other compounds.
2. Mn(II) complex revealed a higher bactericidal effect than other complexes against all tested bacterial strains.
3. The 2,3-diaminopyridine has the lowest antimicrobial activity in comparison with other compounds.
4. Mn(II) complex showed the best antifungal action against the tested fungi.
5. All complexes were more effective towards Gram-positive* bacteria* while less active against Gram-negative bacteria.
6. Low concentrations of 2,3-diaminopyridine did not affect *S. epidermidis* and *Salmonella*sp.
7. In comparison to the common medication ampicillin, 2,4-dihydroxybenzaldehyde, H_2_L ligand, and metal complexes had strong antibacterial activity against *B. cereus* and *S. epidermidis*, exceptfor low quantities of 2,3-diaminopyridine.
8. The antibacterial action of the 2,3-diaminopyridine, 2,4-dihydroxy benzaldehyde, H_2_Lligand, and metal complexes increases as the concentration increases.
9. The 2,4-dihydroxy benzaldehyde and all the complexes displayed higher antifungal activity against *A. solani*compared to the standard drug terconazole.
10. The 2,4-dihydroxybenzaldehyde andMn(II) complex have the best antifungal action against *C. albicans* compared to other compounds and the standard drug terconazole.

**Table 11:** Antibacterial activity of the 2,3-diaminopyridine, 2,4-dihydroxy benzaldehyde, H_2_Lligand, and metal complexes compared to ampicillin.

Antibacterial agent	Concentration, µg/ml	Gram-positive bacteria		Gram-negative bacteria	
		*B. cereus*	*S. epidermidis*	*E. faecalis*	*Salmonella *sp*.*
2,3-Diaminopyridine	50	9 ± 0.14	− ve	13 ± 0.14	− ve
	100	11 ± 0.14	− ve	15 ± 0.14	− ve
	150	14 ± 0.14	5 ± 0.14	17 ± 0.14	6 ± 0.14
2,4-Dihydroxy benzaldehyde	50	28 ± 0	21 ± 0.03	25 ± 0.14	9 ± 0.06
	100	30 ± 0	24 ± 0.03	27 ± 0.06	12 ± 0.03
	150	32 ± 0	26 ± 0.03	29 ± 0.06	15 ± 0
H_2_L ligand	50	10 ± 0	8 ± 0.14	9 ± 0.06	9 ± 0.03
	100	12 ± 0	10 ± 0.06	12 ± 0.06	11 ± 0.03
	150	14 ± 0	12 ± 0.06	14 ± 0.06	13 ± 0.03
Cu(II) complex	50	6 ± 0.03	10 ± 0.03	11 ± 0.03	14 ± 0
	100	9 ± 0.03	12 ± 0.03	13 ± 0.03	16 ± 0
	150	12 ± 0.03	14 ± 0.03	15 ± 0.03	18 ± 0
Co(II) complex	50	10 ± 0.03	12 ± 0.03	13 ± 0	13 ± 0.06
	100	12 ± 0.03	14 ± 0.03	15 ± 0	16 ± 0.03
	150	14 ± 0	16 ± 0.03	17 ± 0	18 ± 0.03
Mn(II) complex	50	23 ± 0	20 ± 0	11 ± 0.06	21 ± 0
	100	25 ± 0	22 ± 0	13 ± 0.06	23 ± 0
	150	27 ± 0	24 ± 0	15 ± 0.06	25 ± 0
Ni(II) complex	50	8 ± 0.03	9 ± 0.06	8 ± 0.14	12 ± 0.03
	100	10 ± 0.03	11 ± 0.06	10 ± 0.14	13 ± 0.03
	150	12 ± 0	13 ± 0.03	12 ± 0.06	15 ± 0.03
Pd(II) complex	50	9 ± 0.03	5 ± 0.14	7 ± 0.14	8 ± 0.14
	100	11 ± 0.03	6 ± 0.03	8 ± 0.14	10 ± 0.14
	150	13 ± 0.03	9 ± 0.03	10 ± 0.14	12 ± 0.14
Ampicillin	50	− ve	− ve	6 ± 0	8 ± 0
	100	− ve	− ve	8 ± 0	10 ± 0
	150	− ve	6 ± 0.14	10 ± 0	12 ± 0

^a^Numbers as given in Scheme 2.

**Table 12:** Antifungal activity of the 2,3-diaminopyridine, 2,4-dihydroxy benzaldehyde, ligand(H_2_L) and metal complexes **(1–5)** compared to terconazole.

Antifungal agent	Concentration, µg/ml	Fungi		
		*A. flavus*	*A. solani*	*C. albicans*
2,3-Diaminopyridine	50	11 ± 0.06	6 ± 0.14	12 ± 0
	100	13 ± 0.06	8 ± 0.14	14 ± 0
	150	15 ± 0.03	10 ± 0.03	16 ± 0
2,4-Dihydroxy benzaldehyde	50	16 ± 0.03	10 ± 0.03	24 ± 0
	100	18 ± 0	12 ± 0.03	26 ± 0
	150	20 ± 0	14 ± 0.03	28 ± 0
H_2_L ligand	50	14 ± 0.03	6 ± 0.03	13 ± 0.03
	100	16 ± 0.03	8 ± 0.03	15 ± 0
	150	18 ± 0.03	10 ± 0.03	17 ± 0
Cu(II) complex	50	16 ± 0.14	12 ± 0.06	10 ± 0.03
	100	18 ± 0.06	14 ± 0.06	12 ± 0.03
	150	20 ± 0.06	16 ± 0.06	14 ± 0.03
Co(II) complex	50	15 ± 0.14	9 ± 0.14	16 ± 0
	100	17 ± 0.14	11 ± 0.06	18 ± 0
	150	19 ± 0.14	13 ± 0.06	20 ± 0
Mn(II) complex	50	23 ± 0.03	16 ± 0.03	26 ± 0
	100	25 ± 0.03	18 ± 0	28 ± 0
	150	27 ± 0.03	20 ± 0	30 ± 0
Ni(II) complex	50	20 ± 0.03	12 ± 0.06	11 ± 0.03
	100	22 ± 0.03	17 ± 0.14	13 ± 0.03
	150	24 ± 0.03	19 ± 0.14	15 ± 0.03
Pd(II) complex	50	16 ± 0.03	11 ± 0.14	11 ± 0.06
	100	18 ± 0.03	13 ± 0.14	13 ± 0.03
	150	20 ± 0.03	15 ± 0.14	15 ± 0.03
Terconazole	50	19 ± 0.14	5 ± 0	18 ± 0
	100	21 ± 0.06	7 ± 0	20 ± 0
	150	23 ± 0.06	10 ± 0	22 ± 0

^a^Numbers as given in Scheme 2.

#### MIC and MMC

**Fig. 15:**
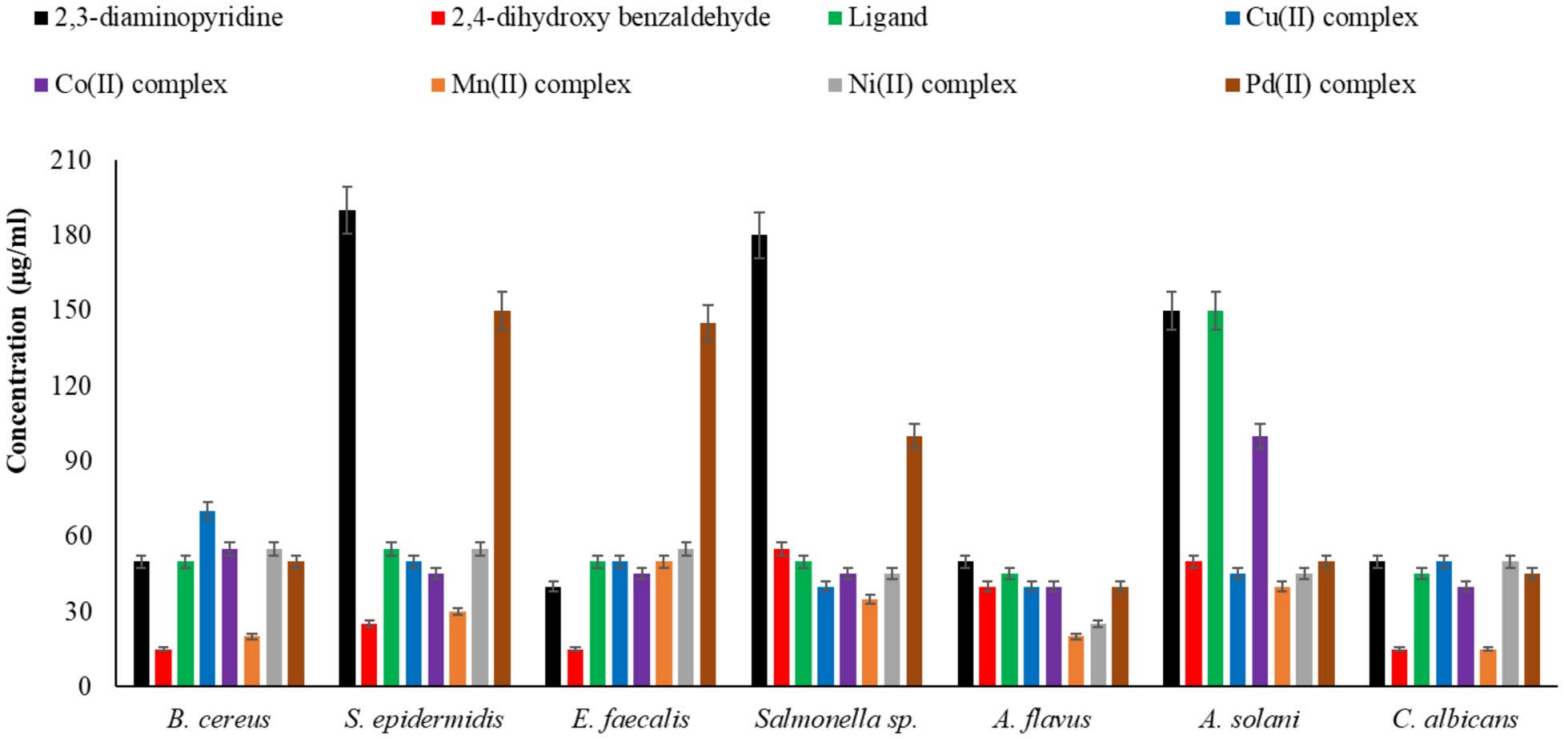
Minimum inhibition concentration (MIC) values of 2,3-diaminopyridine, 2,4-dihydroxybenzaldehyde, ligand (H_2_L), and metal complexes**(1–5)**.

**Fig. 16:**
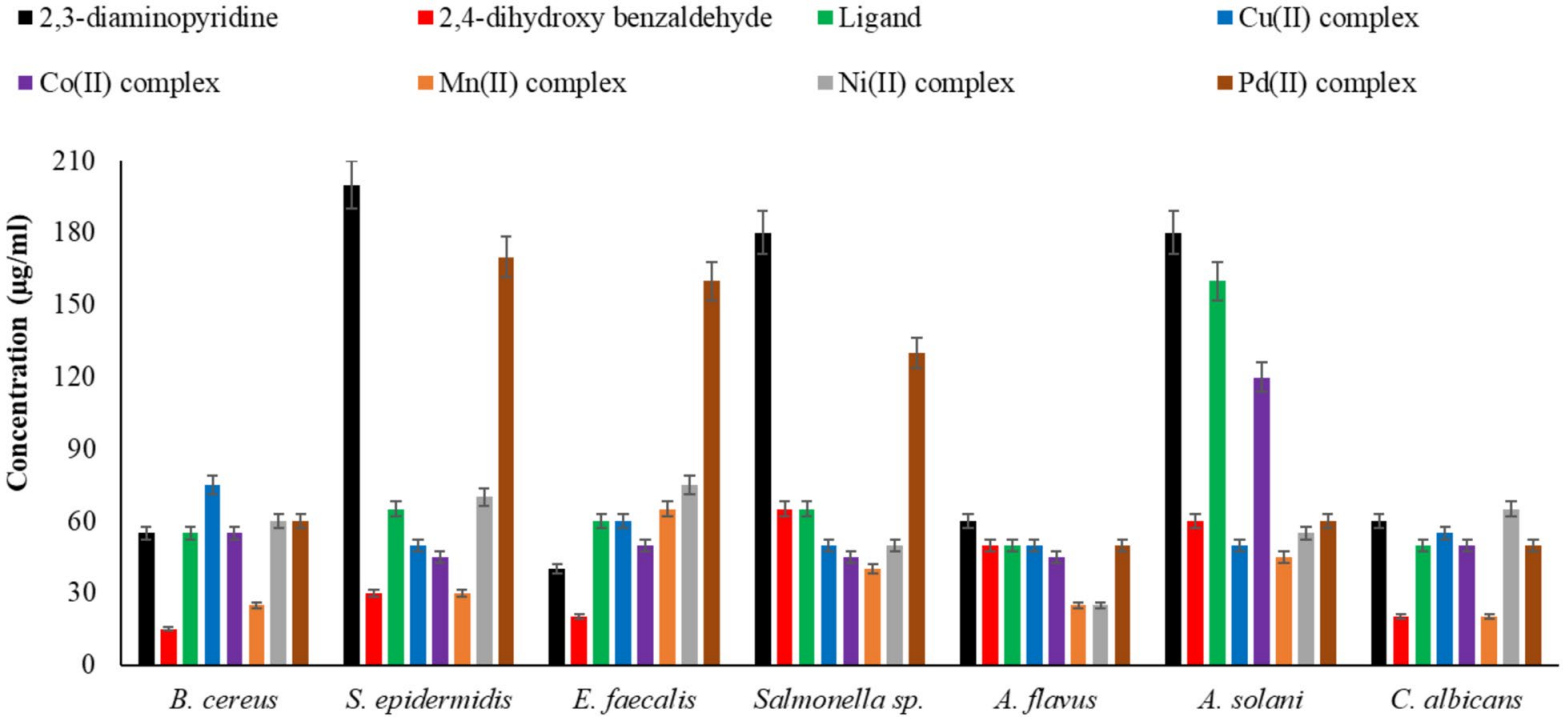
Minimum microbicidal concentration (MMC) values of 2,3-diaminopyridine, 2,4-dihydroxybenzaldehyde, ligand (H_2_L), and metal complexes **(1–5)**.

Assessments of microbial cell viability were also examined throughout the current experiment. The results are shown in Fig. 15. As the lowest dose at which detectable microbial growth is inhibited, MIC values are defined. As the concentration of ligand and its complexes grew, the viability of microbial cells declined for all substances. Lower levels of antibacterial activity are suggested by the 2,3-diaminopyridine’s higher bacterial cell viability. Nevertheless, out of all the studied microbial strains, 2,4-dihydroxy benzaldehyde showed the lowest bacterial cell viability. When compared to ligand and other complexes, the Pd(II) complex showed a poor antibacterial effect but a robust antifungal action against the tested fungal strains, as indicated by its improved bacterial cell viability. When it came to *A. flavus*, *A. solani*, and *C. albicans*, the Mn(II) complex had the strongest antifungal action. The MMC values of 2,3-diaminopyridine, 2,4-dihydroxybenzaldehyde, H_2_L ligand, and metal complexes are displayed in Fig. 16. In contrast to the MMC values of other compounds that were greater than their MIC values, the MMC values of 2,4-dihydroxy benzaldehyde, Mn(II) complex, and their MIC values matched, showing their significant potential as strong antibacterial agents compared to other previous studies (Table 13)^80–85^.

**Table 13:** Minimum inhibitory concentration (MIC) of the ligand and its metal complexes against selected bacterial and fungal strains.

Compound type	*B. cereus*	*Salmonella sp.*	*C. albicans*	Aspergillus sp.	References
H_2_L ligand	50	50	45	45	Present study
2,3-Diaminopyridine-vanillin	64	128	64	128	^80^
2,3-Diaminopyridine	15	55	15	40	Present Study
2,4-Diaminopyridine	12.5	50	12.5	25	^81^
Mn(II) complex	20	35	15	20	Present Study
Mn(II) Schiff Base	25	50	25	40	^82^
Mn(II) tridentate	32	64	16	32	^83^
Pd(II) complex	50	100	45	40	Present Study
	100	200	50	50	^84^
Pd(II) pyridine	125	150	62	62	^85^

When compared to the parent 2,3-diaminopyridine ligand, the metal complexes often showed increased antibacterial activity. Tweedy’s chelation theory explains this increase in bio-efficacy following coordination^86^. This hypothesis states that a delocalization of π-electrons around the whole chelate ring results from the partial sharing of the positive charge of the metal ion with the donor atoms (N and O) of the ligand. The complex becomes more lipophilic as a result of this electronic redistribution, which lessens the polarity of the metal core. According to Overtone’s concept of cell permeability, lipophilic species are more likely to pass through the microbial cell’s lipid-rich semipermeable membrane^87^. These complexes’ enhanced lipophilicity makes it easier for them to get through the cell wall’s lipid bilayer and disrupt essential intracellular functions. Once within the cell, the complexes may attach to DNA or disable cellular enzymes, blocking the microorganism’s regular metabolic processes^88^. In particular, the structure’s phenolic hydroxyl groups and azomethine (> C = N-) connection are crucial; the nitrogen and oxygen atoms offer active sites for hydrogen bonding with the cell’s enzymatic proteins, further impairing their biological activity^89,90^.

The remarkable antifungal potency of the Mn(II) complex, which produced the lowest MIC values (15–20 µg/ml) against *A. flavus*, *A. solani*, and *C. albicans*, is a noteworthy finding in this investigation. This high activity indicates that the Mn(II) center has a particular affinity for fungal enzymatic pathways, potentially those involved in the manufacture of ergosterol. On the other hand, the Pd(II) complex showed a different profile: high MIC values against *S. epidermidis* and *E. faecalis* showed that although it had a strong antifungal effect, its antibacterial efficacy was rather low. The square planar shape of Pd(II), which may encounter steric or electrical hurdles when interacting with the thick peptidoglycan layer of Gram-positive bacteria, may be responsible for this selectivity. However, Pd(II) is still effective against the unique cell wall composition of fungi.

#### The time-kill assay

**Fig. 17:**
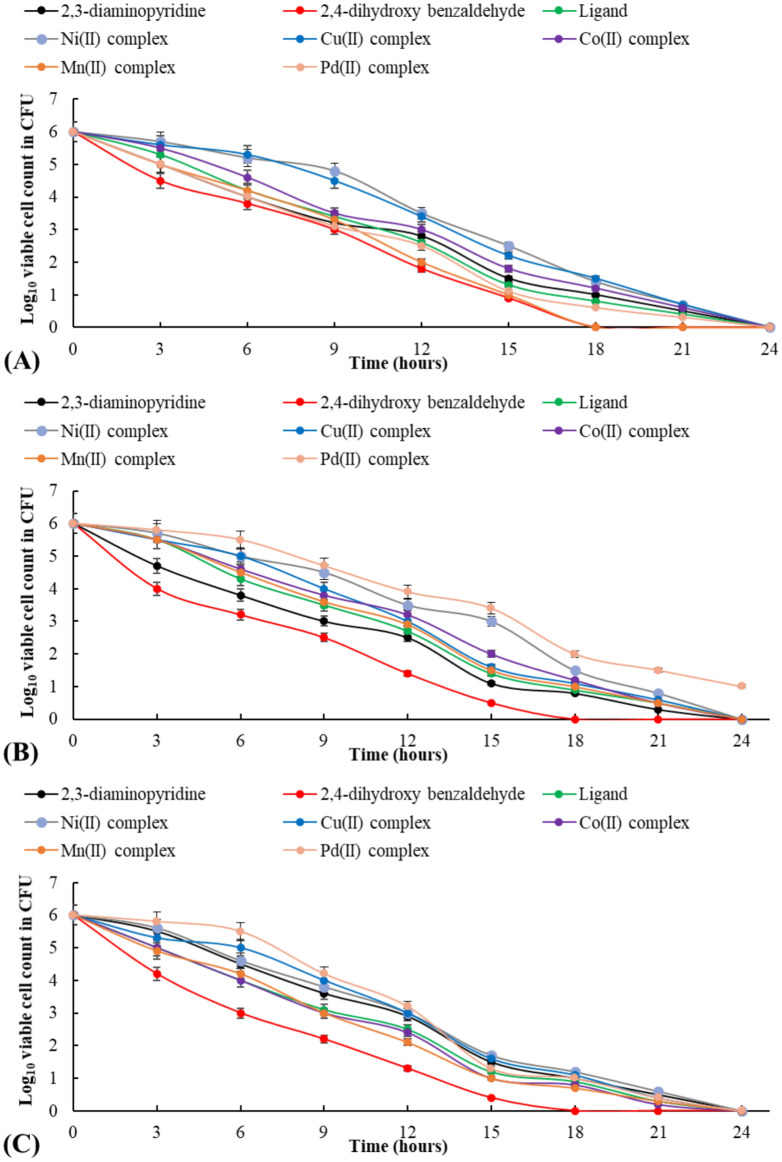
Time-kill assay for *B. cereus* (**A**), *E. faecalis* (**B**), and *C. albicans* (**C**) treated by 2,3-diaminopyridine, 2,4-dihydroxybenzaldehyde, ligand (H_2_L), and metal complexes **(1–5)**.

The time-kill assay for the 2,3-diaminopyridine, 2,4-dihydroxy benzaldehyde, H_2_L ligand, and metal complexes against *C. albicans* as a fungal model, *B. cereus*, and *E. faecalis*as a Gram-positive and Gram-negative bacterium model, respectively, displayed the viable cells in the range of 0to 1 log_10 _CFU/ml throughout 24 h of cultivation (Fig. 17).In contrast, it ranged between 4–4.8 log_10_ CFU/ml at 3 h and 1.3–3.9 log_10_ CFU/ml during 12 h of incubation. It ranged between 0and 1.5 log_10_ CFU/ml at 18 h. Around 9 h for *C. albicans*, *B. cereus*, and *E. faecalis*, the time-kill assay revealed 2,4-dihydroxy benzaldehyde, while Mn(II) complex revealed a 3 log reduction time-kill assay of 9 h for *B. cereus*, and *C. albicans*, and 12 hours or more for *E. faecalis*. Compared to other treatments, it was noted that 24 h were not enough to cause a complete inhibition action for Pd(II) complex against *E. faecalis*. After 18 h, 2,4-dihydroxybenzaldehyde and Mn(II) complex led to killing *B. cereus,* while only 2,4-dihydroxy benzaldehyde killed *E. faecalis* and *C. albicans*.

#### POX and CAT activities

**Fig. 18:**
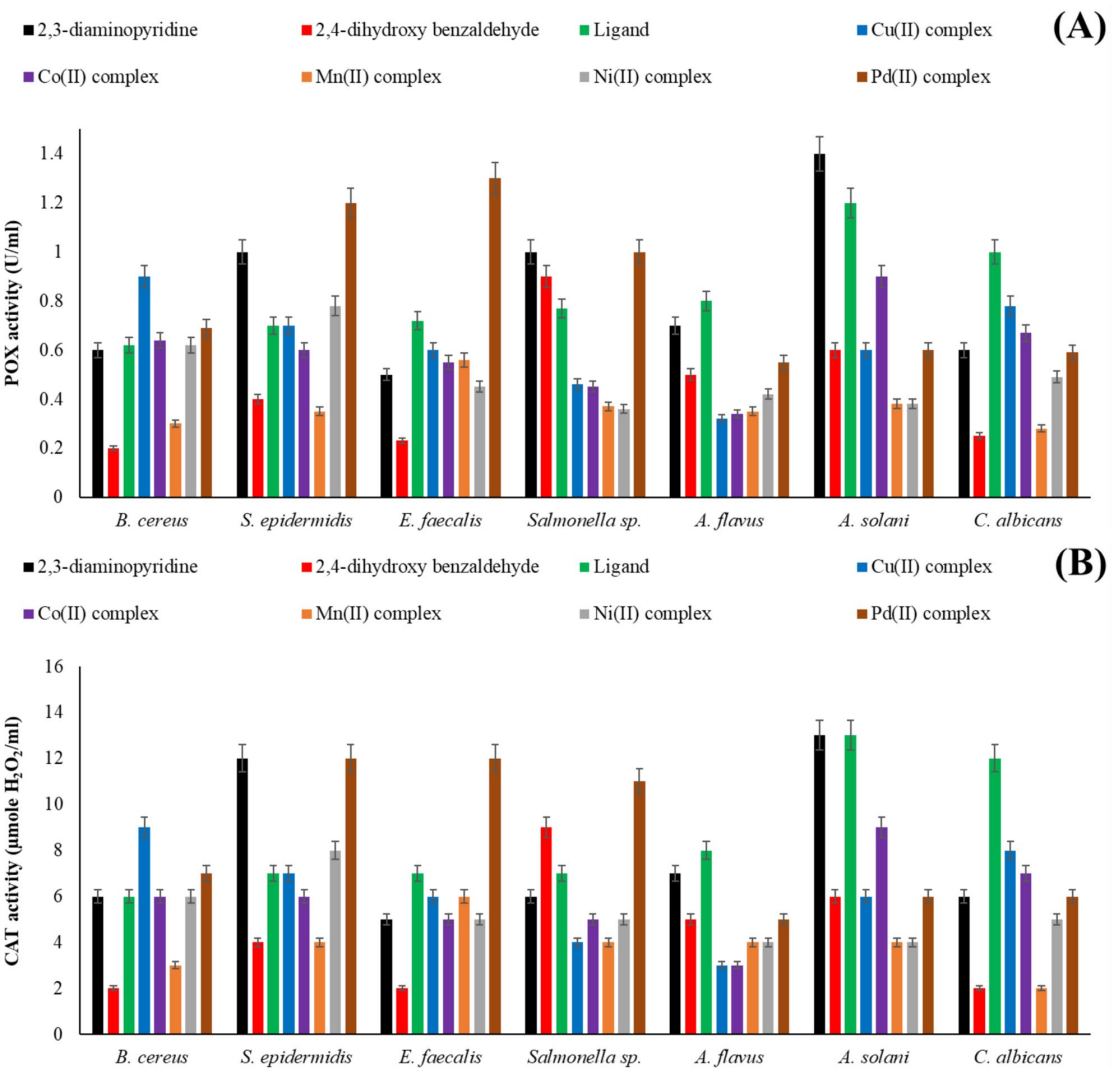
The activity of POX (**A**) and CAT (**B**) for bacterial and fungal strains treated with 2,3-diaminopyridine, 2,4-dihydroxybenzaldehyde, ligand (H_2_L), and metal complexes **(1–5)**.

The antioxidant behavior (POX and CAT) of the 2,3-diaminopyridine, 2,4-dihydroxybenzaldehyde, H_2_L ligand, and metal complexes was evaluated in *A. flavus*, *A. solani*, *C. albicans*, *B. cereus*, *S. epidermidis*, *Salmonella* sp., and *E. faecalis* (Fig. 18). After ligand, 2,3-diaminopyridine, or Pd(II) complex treatment, the microbial strains’ POX and CAT activity varied from mild to powerful in relation to 2,4-dihydroxybenzaldehyde and other metal complexes. Gram-negative bacteria had higher CAT and POX activities than Gram-positive bacteria, indicating their higher resistance to the prepared compounds. This difference might be due to the difference in cell wall structure between the two types of bacteria^79^. Gram-negative bacteria have a multilayered cell wall structure enhanced with high amounts of lipids that might decrease the entrance rate of tested compounds, leading to a decrease in their antimicrobial action, including enzymatic activities. Gram-positive bacteria have a thick and solid cell wall structure enhanced with high amounts of peptidoglycan, which might also decrease the penetration of compounds throughout the cell wall and interaction with the bacterial enzymes. The active functional groups in the compounds are responsible for the bacterial cell wall destruction and have the ability to interfere with the bacterial enzymes’ synthesis^91^.

### Proposed antimicrobial mechanism of action

The significant enhancement in antimicrobial efficacy of the metal complexes relative to the free Schiff base ligand (H_₂_L) and its precursors suggests a synergistic bio-activation upon coordination. As previously mentioned, the proposed mechanism of action can be elucidated through the lens of Tweedy’s chelation theory and Overtone’s permeability concept^86,87^.

#### Enhanced lipophilicity and membrane permeation

According to Overtone’s concept of cell permeability, the lipid membrane surrounding the microbial cell favors the passage of only lipid-soluble materials. In the current study, the free ligand contains polar groups (azomethine and hydroxyl) that limit its lipid solubility. However, upon coordination with transition metal ions [Cu(II), Co(II), Ni(II), Mn(II), and Pd(II)], the positive charge of the metal ion is partially shared with the ONNO donor atoms of the Schiff base. This electronic delocalization reduces the polarity of the central metal atom and increases the lipophilic character of the entire complex. This increased lipophilicity facilitates the penetration of the complex through the microbial lipid bilayer, allowing it to reach intracellular targets more efficiently than the free ligand.

#### Internalization and cellular interference

Once internalized, the complexes likely exert their microbicidal effects through several parallel pathways, such as enzymatic inhibition, at which the metal complexes may compete with natural enzymatic metal binding sites or bind to the sulfhydryl (-SH) groups of vital cellular proteins^91^. This interaction can lead to the denaturation of essential enzymes, thereby arresting metabolic processes as observed in the time-kill assay, which showed a rapid decline in microbial viability. Transition metals, particularly redox-active ions like Cu(II) and Mn(II), can catalyze the formation of reactive oxygen species (ROS) through Fenton-type reactions^5–9^. These radicals (such as OH and O_2_^.-^) induce oxidative damage to cellular lipids (lipid peroxidation) and proteins, compromising the structural integrity of the cell. In addition, the current experimental DNA-binding studies and molecular docking simulations confirm that these complexes have a high affinity for DNA intercalation. By sliding between the nitrogenous base pairs, the complexes stabilize the DNA-drug adduct, effectively inhibiting DNA replication and transcription, which eventually triggers programmed cell death or growth arrest^2^.

#### Correlation with cell wall structure

The variation in activity between Gram-positive (e.g., *B. cereus*) and Gram-negative (e.g., *E. coli*) bacteria further supports a membrane-dependent mechanism^79^. The complex, thick peptidoglycan layer of Gram-positive bacteria, while robust, lacks the outer lipopolysaccharide membrane found in Gram-negative strains. The ability of our synthesized complexes to bridge these structural barriers is a testament to the optimized geometry and electronic properties of the 2,3-diaminopyridine derivative Schiff base framework.

### Cytotoxicity

In recent years, many studies have been done on transition metal ion complexes showing Schiff bases as potential anticancer, antibacterial, and antifungal medications^92^. Evaluating the anticancer effects of these metal complexes and ligand against MCF-7 cells is one of our key research objectives. The MTT test is a commonly used method in cell biology for measuring mitochondrial dehydrogenase activity, which is a proxy for cell viability. At different concentrations (0.01, 0.1, 1, 10, 100 μmol/mL), the testing ligand and its Cu(II), Co(II), Mn(II), Ni(II), and Pd(II) complexes were given to the cells. The outcomes were contrasted with the solvent control (an untreated cell in DMSO) and the positive control (a cell treated with doxorubicin). The IC_50_ was established in order to evaluate the cytotoxicity of the chemical substances that were examined.

**Fig. 19:**
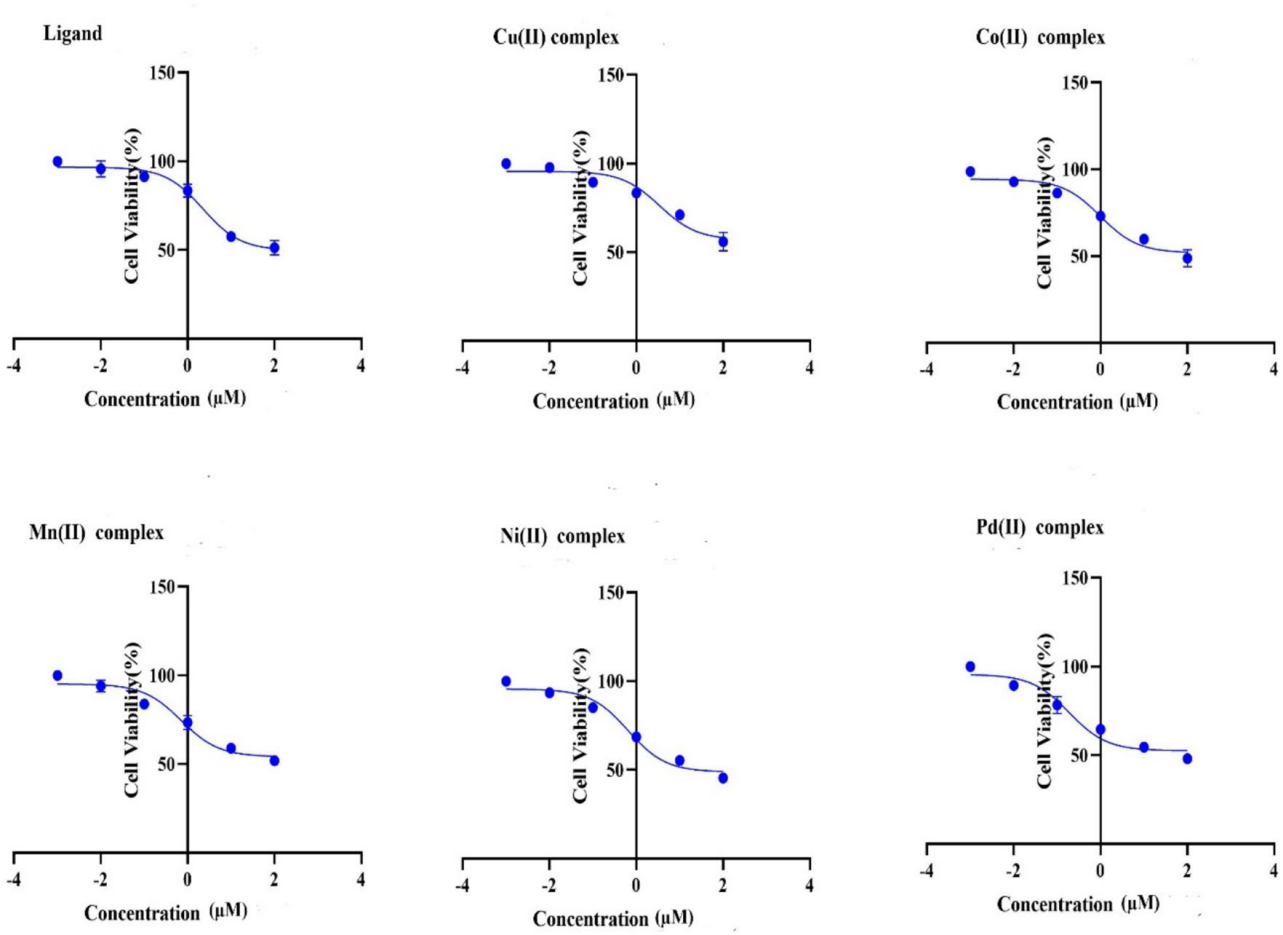
Cell viability curves of the MCF-7 cancer cell line that was treated with the ligand (H_2_L) and metal complexes **(1–5)** with various concentrations for 48 h by MTT assay.

**Fig. 20:**
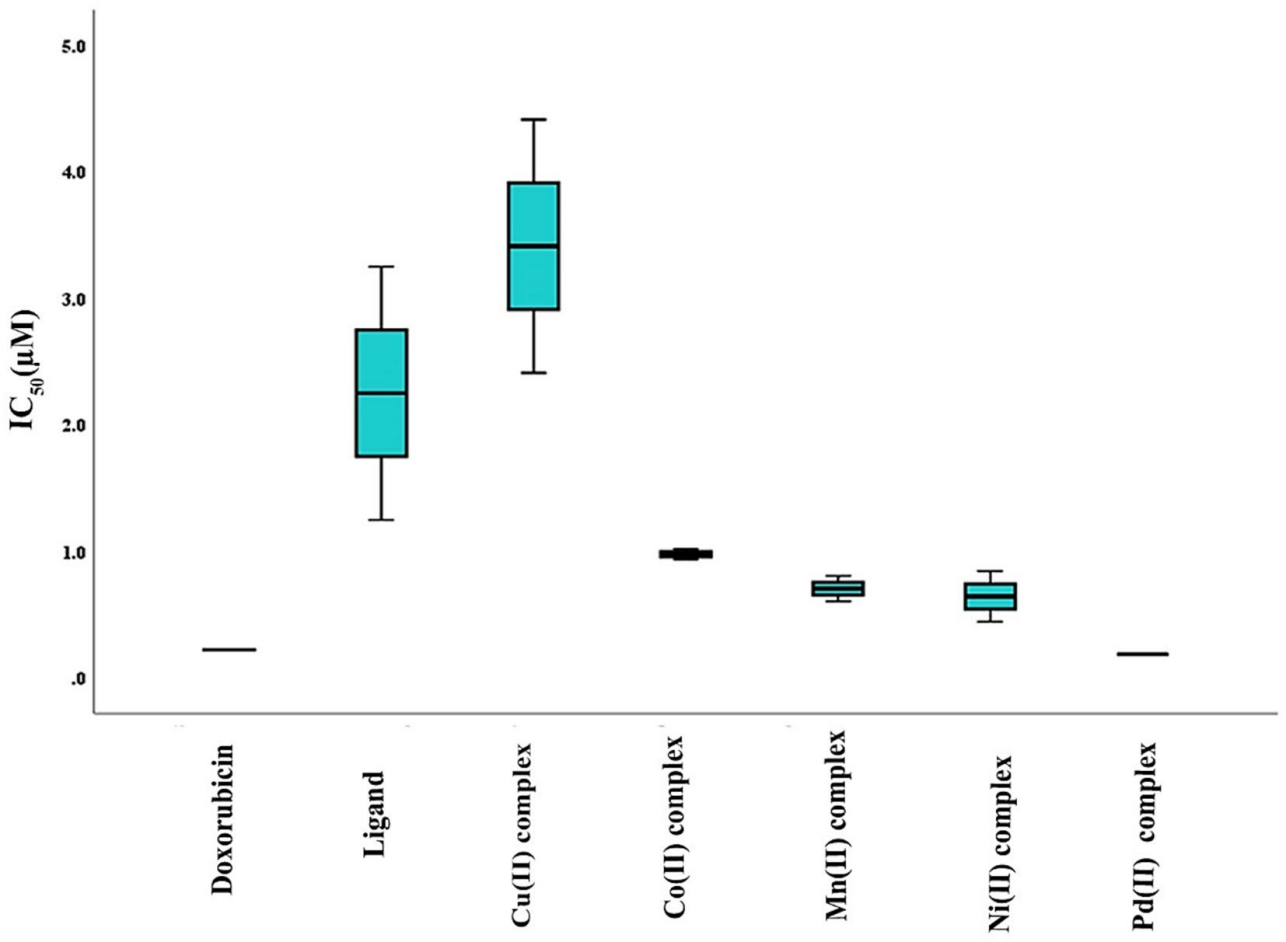
IC_50_ graph of cytotoxicity of the ligand (H_2_L) and metal complexes **(1–5)**.

It was determined that both the ligand and its related complexes had anti-proliferative activity in vitro using the MCF7 cell line. The findings are displayed in Fig. 19, suggesting that these complexes inhibit these cancer cells from growing. Based on their IC_50_ values, all of the complexes show good cytotoxicity when compared to the percentage of viable cells (Table 14 and Fig. 20). Furthermore, the metal complexes typically exhibit greater cytotoxicity in comparison to the corresponding Schiff base ligand^93^. The complexes’ order of cytotoxicity for MCF-7 is complex of Cu(II) < ligand < complex of Co(II) < complex of Mn(II) < complex of Ni(II) < complex of Pd(II). The finding that metal complexes have more pharmacological action than free ligand is correlated with the ligand ability to bind to and break the double helix structure of tumor cell DNA. A plausible explanation is that coordination leads to charge equilibration, which diminishes the polarity of the central metal ion and the ligand, hence facilitating complex penetration through the cell membrane’s lipid layer^94^.

**Table 14:** The IC_50_ of the ligand (H_2_L) and its metal complexes (**1–5**) against the MCF-7 cancer cell line.

Compounds	Cytotoxicity (IC_50_ μmole/mL)
H_2_L ligand	2.251 ± 1.0
Cu(II) complex	3.412 ± 1.02
Co(II) complex	0.98 ± 0.04
Mn(II) complex	0.708 ± 0.1
Ni(II) complex	0.647 ± 0.2
Pd(II) complex	0.189 ± 0.01
Doxorubicin	0.225 ± 0.004

All data presented as mean ± SD, IC_50_; The half inhibitory concentration.

Notably, the most promising results were obtained with the Pd(II) complex, which showed an IC_50_ against MCF-7 cells of 0.189 μmol/mL, which is less than the used standard drug doxorubicin. The Cu(II) complex may appear to be the least active against MCF-7 of all the complexes under study. The DNA binding data and the complexes’ order of cytotoxicity match. The current study’s findings could be consistent with other research that found a significant relationship between anticancer activity and drug–DNA interaction^95,96^. As a result, a hypothesis regarding the enhanced cytotoxic activity of the produced complexes may be connected to their increased DNA-binding affinity. Consequently, these complexes can be regarded as highly promising anticancer treatment agents.

### Correlation analysis

**Fig. 21:**
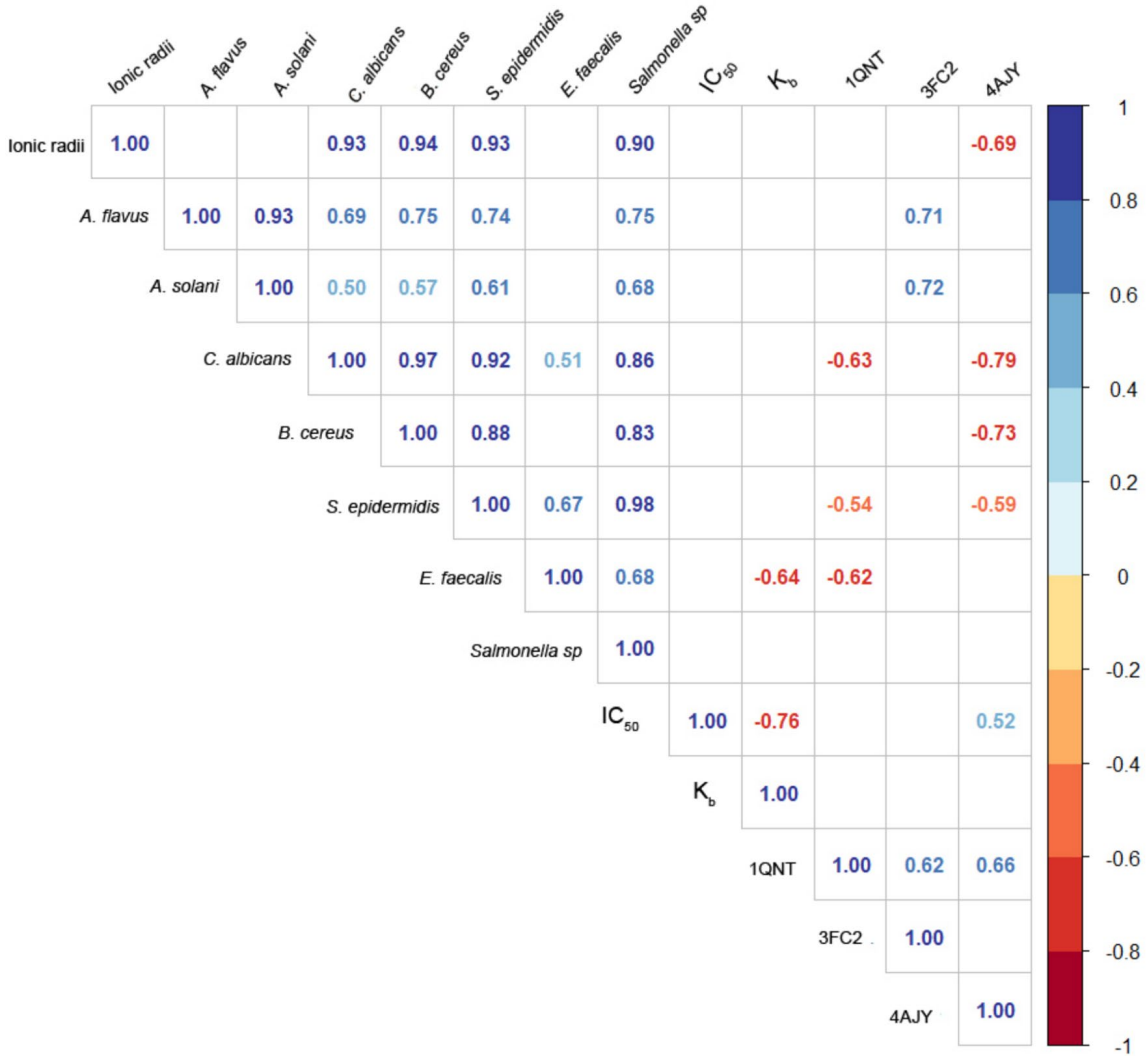
Correlation analysis. Red numbers indicate significant negative correlations, and blue numbers indicate significant positive correlations (*p* < 0.05), and the darker the color, the stronger the correlation.

There is a significant positive correlation between the ionic radius and the antimicrobial action of metal complexes against fungi and bacteria such as* C. albicans, B. cereus, S. epidermidis,* and *Salmonella sp.* (*p* < 0.001). Large ionic radii and higher electronegativity reduce the effective positive charges on the metal complexes, facilitating their interaction with the highly reactive biological membranes. These results may confirm those of AbouElleef et al.^1^, who demonstrated that the high atomic radius and electronegative metal ions in their complexes exhibit high antimicrobial activity. Moreover, it has been demonstrated that the cytotoxicity of complexes (IC_50_) significantly negatively correlates with DNA-binding constants (K_b_) (*p* < 0.001), indicating that complexes with higher DNA-binding affinities are also more cytotoxic against the MCF-7 cell line. Furthermore, the docking results of the interaction of (4AJY) in MCF-7 breast cancer exhibited a significant negative correlation with the ionic radius, *C. albicans, B. cereus, S. epidermidis* (*p* = 0.014,* p* < 0.001, *p* = 0.001, and *p* = 0.01, respectively) besides it had a significant positive correlation with the cytotoxicity of metals IC_50_ (*p* = 0.026). The correlation results made it possible to show how important interactions are for illustrating the tendency towards complexation of the complexes with the biological components in MCF-7 cancer cells, which ultimately leads to their death (Fig. 21 and Table 15).

**Table 15:** Correlation analysis.

		*A. flavus*	*A. solani*	*C. albicans*	*B. cereus*	*S. epidermidis*	*E. faecalis*	*Salmonella sp.*	IC_50_	Kb	1QNT	3FC2	4AJY
Ionic radii	r	0.57	0.42	0.93	0.94	0.93	0.25	0.9	− 0.26	− 0.083	− 0.53	0.21	− 0.69
	*p*	0.052	0.18	** < 0.001**	** < 0.001**	** < 0.001**	0.44	** < 0.001**	0.4	0.79	0.08	0.51	**0.014**
*A. flavus*	r		0.93	0.68	0.75	0.74	0.24	0.75	− 0.38	0.14	0.04	0.71	− 0.39
	*p*		** < 0.001**	**0.002**	** < 0.001**	** < 0.001**	0.34	**0.001**	0.11	0.58	0.87	**0.001**	0.1
*A. solani*	r			0.501	0.57	0.61	0.23	0.68	− 0.29	0.16	0.17	0.72	− 0.095
	*p*			**0.034**	**0.014**	**0.007**	0.37	**0.002**	0.23	0.53	0.49	**0.001**	0.71
*C. albicans*	r				0.97	0.92	0.51	0.86	− 0.32	− 0.14	− 0.63	0.14	− 0.79
	*p*				** < 0.001**	** < 0.001**	**0.03**	** < 0.001**	0.2	0.59	**0.005**	0.58	** < 0.001**
*B. cereus*	r					0.88	0.39	0.83	− 0.33	− 0.049	− 0.47	0.29	− 0.73
	*p*					** < 0.001**	0.11	** < 0.001**	0.19	0.85	0.05	0.23	**0.001**
*S. epidermidis*	r						0.67	0.98	− 0.09	− 0.39	− 0.54	0.19	− 0.59
	*p*						**0.003**	** < 0.001**	0.73	0.107	**0.02**	0.46	**0.01**
*E. faecalis*	r							0.68	0.23	− 0.64	− 0.62	− 0.43	− 0.21
	*p*							**0.003**	0.36	**0.004**	**0.007**	0.074	0.4
*Salmonella sp.*	r								0.05	− 0.44	− 0.46	0.22	− 0.45
	*p*								0.85	0.078	0.066	0.39	0.07
IC_50_	r									− 0.76	0.13	− 0.251	0.52
	*p*									** < 0.001**	0.610	0.32	**0.026**
Kb	r										0.33	0.37	− 0.095
	*p*										0.18	0.13	0.71
QNT	r											0.62	0.66
	*p*											**0.006**	**0.003**
FC2	r												0.052
	*p*												0.84

Significant values are in bold.

## Limitations of the study

While the present study provides a comprehensive physicochemical and biological characterization of the novel Schiff base metal complexes, certain limitations must be acknowledged:

1. Lack of single-crystal X-ray diffraction data: Despite exhaustive attempts using slow evaporation and solvent diffusion techniques in multiple solvent systems, diffraction-quality single crystals could not be obtained due to the microcrystalline nature of the complexes. However, this limitation was mitigated by utilizing indexed powder XRD (using CRYSFIRE and CHEKCELL), which, when combined with spectroscopic and magnetic data, provided a robust structural model.
2. Breadth of cytotoxicity testing: The anticancer evaluation focused on the MCF-7 breast cancer cell line. While the results demonstrate high potency, the current study does not calculate a selectivity index against normal, non-malignant cell lines (such as MCF-10A). Future investigations will focus on the differential toxicity profile to better define the therapeutic window.
3. Mechanistic depth in antimicrobial activity: The antimicrobial study established clear bactericidal and fungicidal efficacy through MIC, MMC, and time-kill assays. However, specific molecular-level mechanisms—such as direct measurement of reactive oxygen species generation or proteomic analysis of membrane-bound proteins—were not conducted. These aspects will be the subject of subsequent mechanistically focused research.
4. In vivo validation**:** The current study is entirely in vitro and in silico. While molecular docking successfully predicted the binding affinity to DNA and proteins, the pharmacokinetic profile (ADME) and systemic toxicity of these complexes in animal models remain to be explored before clinical potential can be fully assessed.

## Conclusion

The metal complexes, which were created from a 1:1 molar ratio by condensation of 2,3-diaminopyridine and 2,4-dihydroxy benzaldehyde, respectively, included Cu(II), Co(II), Ni(II), Mn(II), and Pd(II) as the central metal with 4,4'-[(1*E*,1*`E*)-(pyridine-2,3-diyl)bis(azanylylidene) bis(methanylylidene)bis(benzene-1,3-diol] (H_2_L). Utilizing complementary methods including elemental analysis (C, H, N), FT-IR, magnetic measurements, ^1^H NMR, and molar conductivity, the stoichiometric ratios of the synthesized complexes were calculated. Additionally, they used thermal TG, DNA, powder-XRD, and ESR tests in their physicochemical investigations. The elemental studies revealed that the metal-to-ligand molar ratio of these complexes is 1:1. Furthermore, the room temperature magnetic susceptibility results showed that the Co(II), Ni(II), and Mn(II) complexes have an octahedral (Oh) geometry and are paramagnetic in nature, except Cu(II), which is square planar. Conversely, Pd(II) exhibits diamagnetic behavior and attains stability in square planar locations. In dimethylformamide, all complexes are non-electrolytes, according to the molar conductivity investigations. Spectral findings suggest that the ligand is tetradentate, coupled with Co(II) and Ni(II) ions via two phenolic OH and two azomethine nitrogen. In contrast, Cu(II), Mn(II), and Pd(II) complexes were coordinated by two azomethine nitrogen and two phenolic oxygen with deprotonation of OH groups. During the antimicrobial tests, the ligand and its metal complexes revealed antifungal actions better than their antibacterial activity results. The biological assessment using MIC and MBC assays showed that antimicrobial efficacy is greatly influenced by ligand-to-metal center coordination. With MIC values as low as 15–20 µg/ml, the Mn(II) complex was found to be the most effective antifungal agent against *A. flavus*, *A. solani*, and *C. albicans*. The metal complexes, especially those of Cu(II) and Mn(II), showed improved performance against particular bacterial strains while the starting aldehyde, 2,4-dihydroxybenzaldehyde, maintained high intrinsic antibacterial activity. This phenomenon is strongly supported by Tweedy’s chelation theory and Overtone’s concept. The study of molecular docking found that the screened ligand and Cu(II), Co(II), Ni(II), Mn(II), and Pd(II) complexes can inhibit target proteins and serve as effective therapeutic candidates for developing novel anti-breast cancer medications. The interaction between metal complexes and CT-DNA was successfully established by means of the electronic absorption titration method. The cytotoxicity of the ligand and its complexes was investigated using the breast cancer MCF-7 cell line; the complexes showed increased cytotoxicity. In all biological activities examined, metal complexes showed typically excellent potential when compared to their basic ligand.

## Supplementary Information

Supplementary Information.

## Data Availability

Data for experimental runs are presented in this paper and the supplementary information. The datasets utilized and/or examined in the present investigation are obtainable from the relevant author upon reasonable request.
