# Supplementary material for: Synthesis, calf thymus DNA binding, in-vitro cytotoxicity, molecular docking, and antimicrobial studies of novel metal complexes containing a 2,3-diaminopyridine derivative Schiff base
Source: Sci Rep. 2026 May 27;16:16418. doi: 10.1038/s41598-026-49189-5 (PMC13216323; doi:10.1038/s41598-026-49189-5)
Supplement: Supplementary file 1 — Supplementary Information. [file 41598_2026_49189_MOESM1_ESM.docx]

1. **Experimental**
   1. **Chemicals and reagents**

The chemicals 2,4-dihydroxybenzaldehyde, 2,3-diaminopyridine, Cu(CH_3_COO)_2_⋅ H_2_O, Co(CH_3_COO)_2_⋅ 4H_2_O, Ni(CH_3_COO)_2_. 4H_2_O, Mn(CH_3_COO)_2_· 4H_2_O and PdCl_2_.2H_2_O, dimethylsulfoxide (DMSO), and dimethylformamide (DMF) were acquired from Sigma-Aldrich Chemical Co.

The cell culture medium, which includes Dulbecco's Modified Eagle's Medium (DMEM), fetal bovine serum (FBS), trypsin/EDTA, streptomycin, and amphotericin B, was obtained from Gibco, Thermosientific, Germany. ThermoScientific, Germany's Invitrogen is the source of 3-(4, 5-Dimethylthiazol-2-yl)-2,5-diphenyltetrazolium bromide (MTT). The experiment only used analytical reagent-quality chemicals and solvents; no further purification procedures were carried out.

- 1. **Analytical methods of analysis**

Microanalyses of carbon, hydrogen and nitrogen were carried out at the Microanalytical Center, Cairo University, Egypt, using CHNS-932 (LECO) Vario Elemental Analyzer. The metal content in the complexes was determined by standard methods [15-23]. FT-IR spectra were recorded using a Perkin-Elmer 1650 spectrometer (4000–400 cm^−1^) in KBr discs. Ultraviolet–Visible (UV–Vis) spectra of the complexes were recorded in Nujol solution using a Unicom SP 8800 spectrophotometer in dimethylformamide (DMF) at room temperature. The ^1^H-NMR spectra were obtained with a 500 MHz Jeol FX90 Fourier transform spectrometer with DMSO-d_6_ as the solvent and tetramethylsilane (TMS) as an internal standard reference. The ^13^C-NMR spectra were obtained with a 500 MHz Jeol FX90 Fourier transform spectrometer.

The magnetic moment of the prepared solid complexes was determined at room temperature using the Gouy`s method. Mercury(II) (tetrathiocyanato)cobalt(II), [Hg{Co(SCN)_4_}], was used for the calibration of the Gouy tubes. Diamagnetic corrections were calculated from the values given by Selwood [24] and Pascal’s constants. Magnetic moments were calculated using the equation, µ_eff._ = 2.84 [T_M_^coor.^]^1/2^. Molar conductivities of 10^-3^ M solutions of the solid complexes in DMSO were measured by using Jenway 4010 conductivity meter. The thermogravimetric analysis (TGA) of the solid complexes was carried out from room temperature to 1000 °C using a Shimadzu TG-50H thermal analyzer. Thermal properties were studied from room temperature to 1000 °C at the heating rate of 15 °C/min under dynamic nitrogen atmosphere.

Mass spectra were recorded using MS-5988 GS-MS Hewlett-Packard by the EI technique at 70 eV. X-Ray diffraction analysis of complexes was recorded on X-ray diffractometer analysis in the range of diffraction angle 2θ^o^ = 4–80^o^ with Cu K_α1_-radiation. The applied voltage and the tube current are 40 kV and 30 mA, respectively. The diffraction peaks in powder spectra are indexed and the lattice parameters are determined with the aid of CRYSFIRE computer program. The value of interplanar spacing, d, and Miller indices, hkl, for each diffraction peak are determined by using CHEKCELL program [25].

**Fig. S1.** The mass spectrum of the Cu(II) complex **(1)**.

**Fig. S2.** The mass spectrum of the Ni(II) complex **(4)**.

**Fig. S3.** The mass spectrum of the Pd(II) complex **(5)**.
